# Highly variable penetrance of abnormal phenotypes in embryonic lethal knockout mice

**DOI:** 10.12688/wellcomeopenres.9899.2

**Published:** 2017-02-27

**Authors:** Robert Wilson, Stefan H. Geyer, Lukas Reissig, Julia Rose, Dorota Szumska, Emily Hardman, Fabrice Prin, Christina McGuire, Ramiro Ramirez-Solis, Jacqui White, Antonella Galli, Catherine Tudor, Elizabeth Tuck, Cecilia Icoresi Mazzeo, James C. Smith, Elizabeth Robertson, David J. Adams, Timothy Mohun, Wolfgang J. Weninger

**Affiliations:** 1 The Francis Crick Institute, London, UK; 2 Division of Anatomy, Center for Anatomy & Cell Biology, Medical University of Vienna, Wien, Austria; 3 Wellcome Trust Centre for Human Genetics, Oxford, UK; 4 Wellcome Trust Sanger Institute, Cambridge, UK; 5 Sir William Dunn School of Pathology, University of Oxford, Oxford, UK

**Keywords:** mouse, embryo, phenotype, morphology, high-resolution episcopic microscopy, development, penetrance

## Abstract

**Background:** Identifying genes that are essential for mouse embryonic development and survival through term is a powerful and unbiased way to discover possible genetic determinants of human developmental disorders. Characterising the changes in mouse embryos that result from ablation of lethal genes is a necessary first step towards uncovering their role in normal embryonic development and establishing any correlates amongst human congenital abnormalities.

**Methods:** Here we present results gathered to date in the Deciphering the Mechanisms of Developmental Disorders (DMDD) programme, cataloguing the morphological defects identified from comprehensive imaging of 220 homozygous mutant and 114 wild type embryos from 42 lethal and subviable lines, analysed at E14.5.

**Results:** Virtually all mutant embryos show multiple abnormal phenotypes and amongst the 42 lines these affect most organ systems. Within each mutant line, the phenotypes of individual embryos form distinct but overlapping sets. Subcutaneous edema, malformations of the heart or great vessels, abnormalities in forebrain morphology and the musculature of the eyes are all prevalent phenotypes, as is loss or abnormal size of the hypoglossal nerve.

**Conclusions: **Overall, the most striking finding is that no matter how profound the malformation, each phenotype shows highly variable penetrance within a mutant line. These findings have challenging implications for efforts to identify human disease correlates.

## Introduction

Animal models have long been used as experimental surrogates for investigating the role of individual genes in human development and disease. The remarkable degree of conservation in gene sequence and role that we now know exists across species confirms the validity of this approach and genetic manipulation in the mouse provides a commonly used way to explore gene function. The most ambitious example of this is the attempt coordinated by the International Mouse Phenotyping Consortium (IMPC) to generate a catalogue of gene function, using a systematic approach to phenotyping of individual gene knockouts (KO) that cover the entire mouse genome. In generating KO lines from about one quarter of the total mouse genome so far, these studies have revealed that around one third of all mammalian genes are essential for life ^1– 3^, their removal resulting in embryonic or perinatal lethality. The study of such mutant lines provides a unique opportunity to gain a comprehensive overview of the genetic components regulating normal embryo development and, by inference, the identity of genes whose mutation may cause congenital abnormalities or developmental disease.

Deciphering the Mechanisms of Developmental Disorders (DMDD) is a five year, UK-based programme funded by the Wellcome Trust with the goal of studying 240 embryonic lethal KO lines ^3^. By applying systematic phenotyping methods for homozygous mutant embryos with parallel efforts to identify placental abnormalities and changes in early embryo transcriptome profiles, DMDD offers a foundation for identifying novel genes important for developmental or clinical studies. Here we summarise results to date from detailed examination of homozygous mutant embryos at E14.5 for structural abnormalities.

## Materials and methods

### Embryos

All embryos were produced by the Wellcome Trust Sanger Institute ( https://www.sanger.ac.uk/mouseportal/) as part of the DMDD project ^3^. Gene knockout lines produced as part of a systematic programme coordinated by the International Mouse Phenotyping Consortium ( http://www.mousephenotype.org) were designated lethal if no homozygous mutants were present amongst a minimum of 28 pups at P14 and sub-viable if their proportion fell below 13% of total offspring ^2^. All embryos are obtained from heterozygous intercross independently from the P14 viability call. Embryos were harvested from one or more litters at E14.5, fixed in Bouin’s fixative for 24 hours and stored at 4°C in phosphate buffered saline.

### Generation of digital volume data

Embryos were initially scored for gross abnormalities under a dissection microscope before preparation for 3D imaging. Briefly, embryos were dehydrated in methanol (10% steps until 90%, followed by 95% and 100%; at least 2 hours each) and embedded in methacrylate resin (JB-4, PolySciences) containing eosin B and acridine orange, as previously described ^4– 6^. Within each resin block, the embryo was oriented to ensure transverse sectioning along its longitudinal axis. Resin blocks were allowed to polymerise overnight at room temperature, baked at 90°C for 24–48 hours and then subjected to digital volume data generation using high-resolution episcopic microscopy (HREM) ^7^. HREM data was downsized as appropriate to provide an isotropic voxel size of between 2.5–3 µm, depending on original section thickness.

### Data processing and annotation

12 bit raw greyscale image data was adjusted to optimise tissue visualisation using Photoshop 6 (Adobe). Data visualisation and analysis was performed using software packages Amira 5 (ThermoFisher Scientific) and Osirix, versions 6–8 (Pixmeo). Phenotypes were identified by establishing the precise developmental sub-stage of each embryo and comparing it with stage-matched controls ^8^. Phenotyping was performed according to a standardised and sequential procedure using actual and virtual 2D section stacks, essentially as recently described ^9^. Data from each embryo was independently reviewed by a second anatomist, and any discrepancies resolved by joint agreement. Each phenotype call was assigned to a 3D point within the embryo image data stack. Abnormalities were classified with the Mammalian Phenotype (MP) ontology ^10^, using the most specific MP term that described each defect. 3D volume rendered models were employed for developmental staging from external morphology ^8^.

### Data analysis

In order to facilitate summarising of detailed phenotype annotation data, two subsets of the MP terms closer to the root of the ontology were chosen to provide structured “high” and “intermediate” level overviews of DMDD phenotype data. These MP ontology slims are shown in Table 5 and Table 6 ( Supplementary Table 2 and Supplementary 3 for download). The MP terms assigned during annotation of the embryos were summarised into the categories defined by the DMDD slims using the Map2Slim algorithm ( https://metacpan.org/pod/distribution/go-perl/scripts/map2slim). All the terms of the DMDD slims that map to terms used to annotate mutant and wild type embryo phenotypes are listed in Supplementary Table 1A and Supplementary Table 1B, respectively.

MP annotation terms used to describe the phenotypes of each embryo of a line were normalised to remove duplicate terms, and the terms for each embryo were mapped onto the ontology slims. For each line, a set of the unique slim terms observed for the line was generated and lists were produced of all the embryos from the line falling into each of these high or intermediate level categories. This enabled calculation of a penetrance score for each of the broad slim terms, calculated as a ratio of the number of embryos listed for the slim category to the number of homozygous mutant embryos analysed for the line.

To obtain a global view of the phenotypes detected, the frequency of lines showing each of the broad category slim terms were counted across all the lines analysed. In addition, the incidence of embryos scored for every phenotype category described by the slim terms, and the total number of embryos analysed in lines exhibiting each individual phenotype category was counted.

The total number of lines for each slim term that had a penetrance score between 0–0.24, 0.25–0.49, 0.50–0.74 and 0.75–1.00 was recorded. We calculated the cumulative penetrance score for each slim term as the overall sum of the penetrance scores of every line showing this broad category phenotype. In addition, for each of the penetrance intervals listed above, the sum of the penetrance scores was calculated for the lines falling into these categories.

All plots showing analysis of the data were produced using the R software package, version 3.2.1 (2015-06-18) (The R Foundation for Statistical Computing).

### Use of animals

The care and use of all mice in this study were in accordance with UK Home Office regulations, UK Animals (Scientific Procedures) Act of 1986 (PPL 80/2485) and were approved by the Wellcome Trust Sanger Institute’s Animal Welfare and Ethical Review Body.

## Results

### Size of the study

The data for this study comprises 220 homozygous mutant and 114 wild type E14.5 embryos analysed by the DMDD programme. All data is presented in Supplementary Table 4 and Supplementary Table 5 and is and also available on the DMDD web site ( https://dmdd.org.uk). Embryos were obtained from 42 novel gene knockout lines, 31 classified as lethal and 11 as sub-viable ( Table 1; see also Materials and methods). This corresponds to an average of approximately 5 homozygous mutant embryos for each mutant line, although in practice numbers ranged widely from 1 to 11 as a result of variable breeding efficiency and cost limitations inherent in a large scale screening programme ( Supplementary Figure 1). In total, 1,128,247 transverse section images obtained from the 334 embryos formed the basis for examining embryo structure and with the addition of digital resection of datasets in coronal and sagittal planes, scoring of phenotypes was based on examination of 2,536,659 images.

**Table 1.** List of lethal and subviable lines studied. The gene symbol, Mouse Genome Informatics (MGI) ID for the gene, and allele symbol is listed for each line studied along with the number of homozygous mutant embryos analysed, genetic background and the viability status.

Gene	MGI ID	Allele	P14 homozygous viability	E14.5 homozygous mutant embryos analysed	E14.5 wild type embryos analysed	Genetic Background
*1700067K01Rik*	MGI:1920703	1700067K01Rik<tm2a(KOMP)Wtsi>	Lethal	8	2	C57BL/6N;C57BL/6NTac
*4933434E20Rik*	MGI:1914027	4933434E20Rik<tm1a(EUCOMM)Wtsi>	Lethal	6	3	C57BL/6N;C57BL/6NTac
*Adamts3*	MGI:3045353	Adamts3<tm1b(KOMP)Wtsi>	Lethal	7	3	C57BL/6N;C57BL/6NTac
*Adcy9*	MGI:108450	Adcy9<tm1b(EUCOMM)Wtsi>	Subviable	8	3	C57BL/6N;C57BL/6NTac
*Anks6*	MGI:1922941	Anks6<tm1b(KOMP)Wtsi>	Lethal	2	3	C57BL/6N;C57BL/6NTac
*Atp11a*	MGI:1354735	Atp11a<tm1a(KOMP)Wtsi>	Lethal	5	2	C57BL/6N;C57BL/6NTac
*Brd2*	MGI:99495	Brd2<em2Wtsi>	Lethal	5	3	C57BL/6NTac
*Camsap3*	MGI:1916947	Camsap3<tm1a(EUCOMM)Wtsi>	Subviable	4	3	C57BL/6N;C57BL/6NTac
*Celf4*	MGI:1932407	Celf4<tm1a(EUCOMM)Wtsi>	Lethal	5	3	C57BL/6N;C57BL/6NTac
*Chst11*	MGI:1927166	Chst11<tm1a(KOMP)Wtsi>	Lethal	10	2	C57BL/6N;C57BL/6NTac
*Chtop*	MGI:1913761	Chtop<tm1a(EUCOMM)Wtsi>	Lethal	4	3	C57BL/6N;C57BL/6NTac
*Cir1*	MGI:1914185	Cir1<tm3a(KOMP)Wtsi>	Lethal	3	2	C57BL/6N;C57BL/6NTac
*Cmip*	MGI:1921690	Cmip<tm1a(EUCOMM)Wtsi>	Lethal	10	5	C57BL/6N;C57BL/6NTac
*Col4a3bp*	MGI:1915268	Col4a3bp<tm1a(KOMP)Wtsi>	Subviable	2	3	C57BL/6N;C57BL/6NTac
*Cpt2*	MGI:109176	Cpt2<tm1b(KOMP)Wtsi>	Subviable	6	3	C57BL/6N;C57BL/6NTac
*D930028M14Rik*	MGI:3687343	D930028M14Rik<tm1a(EUCOMM)Wtsi>	Lethal	5	3	C57BL/6N;C57BL/6NTac
*Dbn1*	MGI:1931838	Dbn1<tm1b(KOMP)Wtsi>	Subviable	5	2	C57BL/6N;C57BL/6NTac
*Dhx35*	MGI:1918965	Dhx35<tm1b(EUCOMM)Wtsi>	Lethal	1	2	C57BL/6N;C57BL/6NTac
*Exoc3l2*	MGI:1921713	Exoc3l2<tm1b(KOMP)Wtsi>	Lethal	3	4	C57BL/6N;C57BL/6NTac
*Fam46c*	MGI:1921895	Fam46c<tm1b(KOMP)Wtsi>	Lethal	8	3	C57BL/6N;C57BL/6NTac
*H13*	MGI:95886	H13<tm1b(KOMP)Wtsi>	Lethal	7	3	C57BL/6N;C57BL/6NTac
*Kif1bp*	MGI:1919570	Kif1bp<tm1a(KOMP)Wtsi>	Lethal	3	2	C57BL/6N;C57BL/6NTac
*Mybphl*	MGI:1916003	Mybphl<tm1b(KOMP)Wtsi>	Subviable	3	5	C57BL/6N;C57BL/6NTac
*Npat*	MGI:107605	Npat<tm1b(EUCOMM)Wtsi>	Lethal	1	1	C57BL/6N;C57BL/6NTac
*Nsun2*	MGI:107252	Nsun2<tm1a(EUCOMM)Wtsi>	Subviable	6	2	C57BL/6Brd-Tyr<c-Brd>; C57BL/6Dnk; C57BL/6N;C57BL/6NTac
*Nxn*	MGI:109331	Nxn<tm1b(EUCOMM)Wtsi>	Lethal	3	3	C57BL/6N;C57BL/6NTac
*Otud7b*	MGI:2654703	Otud7b<tm1b(EUCOMM)Wtsi>	Lethal	1	3	C57BL/6N;C57BL/6NTac
*Pdzk1*	MGI:1928901	Pdzk1<tm2b(EUCOMM)Wtsi>	Subviable	9	3	C57BL/6N;C57BL/6NTac
*Polb*	MGI:97740	Polb<tm1a(KOMP)Wtsi>	Lethal	6	1	C57BL/6N;C57BL/6NTac
*Prrc2b*	MGI:1923304	Prrc2b<tm1a(EUCOMM)Wtsi>	Lethal	9	4	C57BL/6N;C57BL/6NTac
*Psph*	MGI:97788	Psph<tm1a(EUCOMM)Hmgu>	Lethal	8	3	C57BL/6N;C57BL/6NTac
*Pth1r*	MGI:97801	Pth1r<tm1a(EUCOMM)Hmgu>	Lethal	3	3	C57BL/6N;C57BL/6NTac
*Rundc1*	MGI:2144506	Rundc1<tm1b(EUCOMM)Wtsi>	Subviable	4	1	C57BL/6N;C57BL/6NTac
*Sh3pxd2a*	MGI:1298393	Sh3pxd2a<tm1b(EUCOMM)Wtsi>	Lethal	11	2	C57BL/6N;C57BL/6NTac
*Slc25a20*	MGI:1928738	Slc25a20<tm1a(EUCOMM)Wtsi>	Lethal	6	4	C57BL/6N;C57BL/6NTac
*Slc5a7*	MGI:1927126	Slc5a7<tm1a(KOMP)Wtsi>	Lethal	3	3	C57BL/6N;C57BL/6NTac
*Smg9*	MGI:1919247	Smg9<tm1b(EUCOMM)Wtsi>	Lethal	6	3	C57BL/6N;C57BL/6NTac
*Smpd4*	MGI:1924876	Smpd4<tm2b(KOMP)Wtsi>	Subviable	3	1	C57BL/6N;C57BL/6NTac
*Ssr2*	MGI:1913506	Ssr2<tm1b(EUCOMM)Wtsi>	Lethal	3	0	C57BL/6N;C57BL/6NTac
*Tcf7l2*	MGI:1202879	Tcf7l2<tm1a(EUCOMM)Wtsi>	Lethal	5	4	C57BL/6N;C57BL/6NTac
*Traf6*	MGI:108072	Traf6<tm2a(EUCOMM)Wtsi>	Lethal	9	5	C57BL/6N;C57BL/6NTac
*Unk*	MGI:2442456	Unk<tm1a(KOMP)Wtsi>	Subviable	5	2	C57BL/6N;C57BL/6NTac

### Incidence of structural abnormalities in homozygous mutant embryos

**Figure 1.**
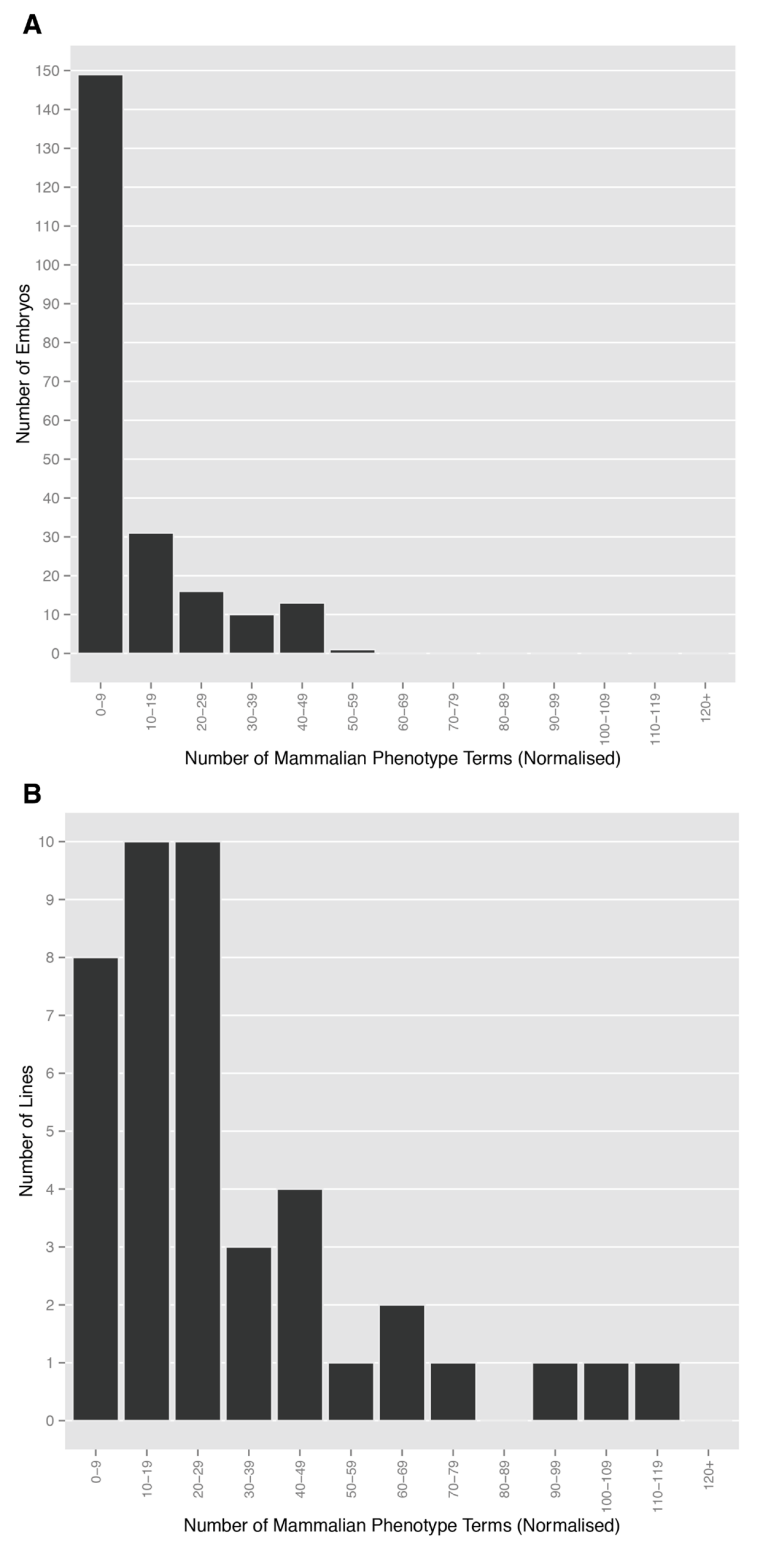
Multiple abnormalities are evident in homozygous mutant embryos. The Mammalian Phenotype Ontology terms scored for ( **A**) each embryo, and ( **B**) each line were normalised to remove duplicate ontology terms. The number of distinct phenotypes scored that fell into categories with a window width of 10 were plotted to show the total number of embryos and lines respectively in each category.

Almost all mutant embryos studied (209/220) showed structural abnormalities that could be identified by a phenotyping procedure previously refined from pilot studies ^9^. The remaining 11 apparently normal embryos were obtained from 9 different lines, each of which yielded several other homozygous mutants bearing detectable morphological abnormalities. We have previously reported that the resolution afforded by 3D datasets obtained by HREM imaging allowed the detection of phenotypic abnormalities spanning in size range from individual nerves and blood vessels to gross organ and tissue malformations ^9^. In the present study, a total of 398 different MP terms were employed to record a total of 2,939 detected mutant embryo phenotypes ( Table 2A and Supplementary Table 1A and Supplementary Table 4). Multiple abnormalities were scored in virtually all homozygous mutant embryos. Most showed up to 10, but in some embryos as many as 50 phenotypes were recorded ( Figure 1A). Whilst a few phenotypes (for example those affecting different parts of vertebrae or different regions of the vertebral column) were often scored repeatedly within affected embryos, their incidence was insufficient to have a significant impact on the overall distribution of phenotype numbers scored per embryo across the whole study. When analysed by individual mutant line, the incidence of detectable abnormalities is more broadly distributed, with more than half of the 42 lines showing between 10 and 49 different phenotypes ( Figure 1B).

**Table 2A.** Frequency of phenotypes identified in homozygous mutant embryos. The Mammalian Phenotype Ontology terms describing phenotypes observed in each embryo were normalised to remove duplicates and the list then ranked in descending order by frequency of embryos exhibiting each phenotype.

MP ID	MP term	Frequency
MP:0013848	subcutaneous edema	64
MP:0004613	fusion of vertebral arches	61
MP:0010418	perimembraneous ventricular septal defect	49
MP:0000783	abnormal forebrain morphology	47
MP:0003686	abnormal eye muscle morphology	45
MP:0001015	small superior cervical ganglion	45
MP:0010420	muscular ventricular septal defect	41
MP:0013835	absent hypoglossal nerve	37
MP:0003826	abnormal Mullerian duct morphology	33
MP:0014021	heterochrony	33
MP:0004269	abnormal optic cup morphology	32
MP:0014001	abnormal vertebral artery topology	32
MP:0013836	abnormal hypoglossal nerve topology	30
MP:0013876	absent ductus venosus valve	29
MP:0000284	double outlet right ventricle	29
MP:0004666	absent stapedial artery	28
MP:0013971	blood in lymph vessels	27
MP:0000703	abnormal thymus morphology	26
MP:0014000	anastomosis between internal carotid artery and basilar artery	25
MP:0000602	enlarged liver sinusoidal spaces	25
MP:0013969	reduced sympathetic cervical ganglion size	25
MP:0008923	thoracoschisis	25
MP:0004163	abnormal adenohypophysis morphology	24
MP:0002237	abnormal nasal cavity morphology	20
MP:0013986	abnormal vitelline vein topology	20
MP:0013967	abnormal infrahyoid muscle connection	18
MP:0004463	basisphenoid bone foramen	18
MP:0008128	abnormal brain internal capsule morphology	16
MP:0000282	abnormal interatrial septum morphology	16
MP:0004268	abnormal optic stalk morphology	16
MP:0013936	abnormal thymus topology	16
MP:0014017	abnormal Wolffian duct connection	15
MP:0013877	abnormal ductus venosus valve morphology	15
MP:0002239	abnormal nasal septum morphology	15
MP:0000497	abnormal small intestine placement	15
MP:0000111	cleft palate	15
MP:0013859	abnormal vitelline vein connection	14
MP:0013826	absent hypoglossal canal	14
MP:0013840	absent segment of posterior cerebral artery	14
MP:0013875	trigeminal neuroma	14
MP:0010496	abnormal pectinate muscle morphology	13
MP:0013834	thin hypoglossal nerve	13
MP:0003827	abnormal Wolffian duct morphology	12
MP:0013842	ductus venosus stenosis	12
MP:0010912	herniated liver	12
MP:0013968	multiple persisting craniopharyngeal ducts	12
MP:0011361	pelvic kidney	12
MP:0010572	persistent right dorsal aorta	12
MP:0002633	persistent truncus arteriosis	12
MP:0013931	abnormal olfactory bulb position	11
MP:0011683	dual inferior vena cava	11
MP:0000914	exencephaly	11
MP:0002169	no abnormal phenotype detected	11
MP:0000154	rib fusion	11
MP:0000161	scoliosis	11
MP:0004110	transposition of great arteries	11
MP:0012303	umbilical vein stenosis	11
MP:0008922	abnormal cervical rib	10
MP:0009917	abnormal hyoid bone body morphology	10
MP:0009770	abnormal optic chiasm morphology	10
MP:0013844	abnormal perichondrial ossification	10
MP:0003345	decreased rib number	10
MP:0011493	double ureter	10
MP:0000445	short snout	10
MP:0002951	small thyroid gland	10
MP:0013878	abnormal ductus venosus valve topology	9
MP:0000841	abnormal hindbrain morphology	9
MP:0010490	abnormal inferior vena cava valve morphology	9
MP:0010853	abnormal lung position or orientation	9
MP:0000141	abnormal vertebral body morphology	9
MP:0002243	abnormal vomeronasal organ morphology	9
MP:0013970	absent connection between subcutaneous lymph vessels and lymph sac	9
MP:0011667	double outlet right ventricle with atrioventricular septal defect	9
MP:0014019	embryo cyst	9
MP:0013977	symmetric azygos veins	9
MP:0002092	abnormal eye morphology	8
MP:0014023	abnormal intestine placement	8
MP:0001303	abnormal lens morphology	8
MP:0000632	abnormal pineal gland morphology	8
MP:0010602	abnormal pulmonary valve cusp morphology	8
MP:0013985	abnormal umbilical vein topology	8
MP:0013965	abnormally deep median sulcus of tongue	8
MP:0010484	bicuspid aortic valve	8
MP:0004646	decreased cervical vertebrae number	8
MP:0013915	abnormal brachial plexus formation	7
MP:0010436	abnormal coronary sinus morphology	7
MP:0000819	abnormal olfactory bulb morphology	7
MP:0009570	abnormal right lung morphology	7
MP:0003078	aphakia	7
MP:0003584	bifid ureter	7
MP:0013949	fusion of axis and occipital bones	7
MP:0013846	retropharyngeal edema	7
MP:0013847	retropleural edema	7
MP:0000153	rib bifurcation	7
MP:0002191	abnormal artery morphology	6
MP:0000079	abnormal basioccipital bone morphology	6
MP:0000788	abnormal cerebral cortex morphology	6
MP:0013995	abnormal external carotid artery origin	6
MP:0013845	abnormal eye muscle topology	6
MP:0002858	abnormal posterior semicircular canal morphology	6
MP:0000759	abnormal skeletal muscle morphology	6
MP:0013871	abnormal stapedial artery topology	6
MP:0001146	abnormal testis morphology	6
MP:0000681	abnormal thyroid gland morphology	6
MP:0004599	abnormal vertebral arch morphology	6
MP:0013996	abnormal vertebral artery origin	6
MP:0013849	absent abducens nerve	6
MP:0000520	absent kidney	6
MP:0009725	absent lens vesicle	6
MP:0006093	arteriovenous malformation	6
MP:0010412	atrioventricular septal defect	6
MP:0013932	fragmented Meckel's cartilage	6
MP:0000963	fused dorsal root ganglion	6
MP:0005157	holoprosencephaly	6
MP:0000480	increased rib number	6
MP:0013992	persistent dorsal ophthalmic artery	6
MP:0013952	retro-esophageal left subclavian artery	6
MP:0004160	retroesophageal right subclavian artery	6
MP:0004158	right aortic arch	6
MP:0020301	short tongue	6
MP:0002989	small kidney	6
MP:0013852	abnormal Mullerian duct topology	5
MP:0010595	abnormal aortic valve cusp morphology	5
MP:0000297	abnormal atrioventricular cushion morphology	5
MP:0013186	abnormal basilar artery morphology	5
MP:0002152	abnormal brain morphology	5
MP:0013874	abnormal ductus venosus topology	5
MP:0013945	abnormal elbow joint morphology	5
MP:0000559	abnormal femur morphology	5
MP:0006063	abnormal inferior vena cava morphology	5
MP:0002135	abnormal kidney morphology	5
MP:0001879	abnormal lymphatic vessel morphology	5
MP:0005236	abnormal olfactory nerve morphology	5
MP:0000150	abnormal rib morphology	5
MP:0004539	absent maxilla	5
MP:0003451	absent olfactory bulb	5
MP:0001014	absent superior cervical ganglion	5
MP:0014003	additional anastomosis between intracranial vertebral arteries	5
MP:0012548	myelocele	5
MP:0000273	overriding aortic valve	5
MP:0000964	small dorsal root ganglion	5
MP:0000694	spleen hypoplasia	5
MP:0013928	thin motoric part of trigeminal nerve	5
MP:0002199	abnormal brain commissure morphology	4
MP:0006065	abnormal heart position or orientation	4
MP:0002249	abnormal larynx morphology	4
MP:0009820	abnormal liver vasculature morphology	4
MP:0005105	abnormal middle ear ossicle morphology	4
MP:0004164	abnormal neurohypophysis morphology	4
MP:0013994	abnormal parasellar internal carotid artery branch morphology	4
MP:0000633	abnormal pituitary gland morphology	4
MP:0013980	abnormal pulmonary artery origin	4
MP:0011655	abnormal systemic artery morphology	4
MP:0011513	abnormal vertebral artery morphology	4
MP:0013855	absent celiac artery	4
MP:0013833	absent olfactory nerve	4
MP:0013362	absent pineal gland	4
MP:0014006	absent posterior communicating artery	4
MP:0013913	absent rib-vertebral column attachment	4
MP:0004846	absent skeletal muscle	4
MP:0004603	absent vertebral arch	4
MP:0010440	anomalous pulmonary venous connection	4
MP:0010530	cerebral arteriovenous malformation	4
MP:0010589	common truncal valve	4
MP:0003924	diaphragmatic hernia	4
MP:0003253	dilated bile duct	4
MP:0013879	duplication of ductus venosus	4
MP:0008534	enlarged fourth ventricle	4
MP:0004612	fusion of vertebral bodies	4
MP:0001914	hemorrhage	4
MP:0003262	intestinal/bowel diverticulum	4
MP:0010404	ostium primum atrial septal defect	4
MP:0013917	persistent right 6th pharyngeal arch artery	4
MP:0000562	polydactyly	4
MP:0001088	small nodose ganglion	4
MP:0013827	thin oculomotor nerve	4
MP:0013858	abnormal azygos vein topology	3
MP:0002928	abnormal bile duct morphology	3
MP:0008026	abnormal brain white matter morphology	3
MP:0004607	abnormal cervical atlas morphology	3
MP:0000820	abnormal choroid plexus morphology	3
MP:0013873	abnormal ductus venosus morphology	3
MP:0010439	abnormal hepatic vein morphology	3
MP:0000823	abnormal lateral ventricle morphology	3
MP:0000598	abnormal liver morphology	3
MP:0000897	abnormal midbrain morphology	3
MP:0013861	abnormal pancreas topology	3
MP:0000613	abnormal salivary gland morphology	3
MP:0013943	abnormal ureter topology	3
MP:0001100	abnormal vagus ganglion morphology	3
MP:0014002	absent extracranial vertebral artery segment	3
MP:0013929	absent eye muscles	3
MP:0003722	absent ureter	3
MP:0000138	absent vertebrae	3
MP:0000640	adrenal gland hypoplasia	3
MP:0005262	coloboma	3
MP:0010433	double inlet heart left ventricle	3
MP:0001785	edema	3
MP:0000274	enlarged heart	3
MP:0006203	eye hemorrhage	3
MP:0005244	hemopericardium	3
MP:0013843	hepatic portal vein stenosis	3
MP:0011659	interrupted aortic arch, type b	3
MP:0013948	intraembryonal intestine elongation	3
MP:0013963	jugular vein stenosis	3
MP:0000692	small spleen	3
MP:0001093	small trigeminal ganglion	3
MP:0013828	thin facial nerve	3
MP:0004057	thin myocardium compact layer	3
MP:0003617	urinary bladder hypoplasia	3
MP:0013851	abnormal Wolffian duct topology	2
MP:0013857	abnormal abdominal muscle morphology	2
MP:0004113	abnormal aortic arch morphology	2
MP:0002747	abnormal aortic valve morphology	2
MP:0004181	abnormal carotid artery morphology	2
MP:0013978	abnormal carotid artery origin	2
MP:0013975	abnormal coronary sinus connection	2
MP:0002279	abnormal diaphragm morphology	2
MP:0013815	abnormal digastric muscle morphology	2
MP:0013865	abnormal dorsal pancreas topology	2
MP:0000961	abnormal dorsal root ganglion morphology	2
MP:0013950	abnormal dorsal root ganglion topology	2
MP:0006011	abnormal endolymphatic duct morphology	2
MP:0013918	abnormal endolymphatic sac topology	2
MP:0006033	abnormal external auditory canal morphology	2
MP:0000266	abnormal heart morphology	2
MP:0003056	abnormal hyoid bone morphology	2
MP:0013966	abnormal infrahyoid muscle morphology	2
MP:0000489	abnormal large intestine morphology	2
MP:0008986	abnormal liver parenchyma morphology	2
MP:0001175	abnormal lung morphology	2
MP:0000458	abnormal mandible morphology	2
MP:0003632	abnormal nervous system morphology	2
MP:0001330	abnormal optic nerve morphology	2
MP:0002177	abnormal outer ear morphology	2
MP:0000492	abnormal rectum morphology	2
MP:0002428	abnormal semicircular canal morphology	2
MP:0002746	abnormal semilunar valve morphology	2
MP:0000496	abnormal small intestine morphology	2
MP:0005107	abnormal stapes morphology	2
MP:0003230	abnormal umbilical artery morphology	2
MP:0002725	abnormal vein morphology	2
MP:0009707	absent external auditory canal	2
MP:0013987	absent intrahepatic inferior vena cava segment	2
MP:0009771	absent optic chiasm	2
MP:0013999	absent parasellar internal carotid artery	2
MP:0013809	absent pectinate muscle	2
MP:0004571	absent vagus nerve	2
MP:0000140	absent vertebral pedicles	2
MP:0003130	anal atresia	2
MP:0010463	aorta stenosis	2
MP:0004055	atrium hypoplasia	2
MP:0010406	common atrium	2
MP:0003586	dilated ureter	2
MP:0013981	double lumen aortic arch	2
MP:0014018	embryo tumor	2
MP:0010200	enlarged lymphatic vessel	2
MP:0008536	enlarged third ventricle	2
MP:0002015	epithelioid cysts	2
MP:0004201	fetal growth retardation	2
MP:0010977	fused right lung lobes	2
MP:0010728	fusion of atlas and occipital bones	2
MP:0013982	inverse situs of great intrathoracic arteries	2
MP:0010647	left atrium hypoplasia	2
MP:0000600	liver hypoplasia	2
MP:0000618	small salivary gland	2
MP:0001102	small superior vagus ganglion	2
MP:0000706	small thymus	2
MP:0011249	abdominal situs inversus	1
MP:0000639	abnormal adrenal gland morphology	1
MP:0010592	abnormal atrioventricular septum morphology	1
MP:0002745	abnormal atrioventricular valve morphology	1
MP:0001614	abnormal blood vessel morphology	1
MP:0000494	abnormal cecum morphology	1
MP:0013862	abnormal cecum position	1
MP:0010744	abnormal cervical flexure morphology	1
MP:0003048	abnormal cervical vertebrae morphology	1
MP:0009495	abnormal common bile duct morphology	1
MP:0012729	abnormal common carotid artery morphology	1
MP:0013930	abnormal digastric muscle connection	1
MP:0004252	abnormal direction of heart looping	1
MP:0014022	abnormal duodenum topology	1
MP:0013924	abnormal dural venous sinus morphology	1
MP:0013927	abnormal facial nerve topology	1
MP:0006107	abnormal fetal atrioventricular canal morphology	1
MP:0000828	abnormal fourth ventricle morphology	1
MP:0005084	abnormal gallbladder morphology	1
MP:0003105	abnormal heart atrium morphology	1
MP:0003922	abnormal heart right atrium morphology	1
MP:0013814	abnormal hepatic portal vein connection	1
MP:0013853	abnormal hepatic portal vein formation	1
MP:0010668	abnormal hepatic portal vein morphology	1
MP:0013973	abnormal hepatic vein connection	1
MP:0005296	abnormal humerus morphology	1
MP:0009913	abnormal hyoid bone greater horn morphology	1
MP:0013824	abnormal hypoglossal canal morphology	1
MP:0002859	abnormal inner ear canal fusion	1
MP:0009804	abnormal interventricular foramen morphology	1
MP:0000281	abnormal interventricular septum morphology	1
MP:0000477	abnormal intestine morphology	1
MP:0013976	abnormal left vena cava superior connection	1
MP:0004881	abnormal lung size	1
MP:0013841	abnormal lymphatic vessel topology	1
MP:0003792	abnormal major salivary gland morphology	1
MP:0000455	abnormal maxilla morphology	1
MP:0000452	abnormal mouth morphology	1
MP:0002108	abnormal muscle morphology	1
MP:0004056	abnormal myocardium compact layer morphology	1
MP:0005269	abnormal occipital bone morphology	1
MP:0013818	abnormal oral cavity morphology	1
MP:0014011	abnormal ovary tissue architecture	1
MP:0004509	abnormal pelvic girdle bone morphology	1
MP:0002748	abnormal pulmonary valve morphology	1
MP:0009571	abnormal right lung accessory lobe morphology	1
MP:0009688	abnormal spinal cord central canal morphology	1
MP:0008023	abnormal styloid process morphology	1
MP:0013979	abnormal subclavian artery origin	1
MP:0001011	abnormal superior cervical ganglion morphology	1
MP:0000787	abnormal telencephalon morphology	1
MP:0005272	abnormal temporal bone morphology	1
MP:0000826	abnormal third ventricle morphology	1
MP:0002368	abnormal thymus capsule morphology	1
MP:0002282	abnormal trachea morphology	1
MP:0001065	abnormal trigeminal nerve morphology	1
MP:0010667	abnormal umbilical vein morphology	1
MP:0000534	abnormal ureter morphology	1
MP:0013925	abnormal vascular plexus formation	1
MP:0000137	abnormal vertebrae morphology	1
MP:0005274	abnormal viscerocranium morphology	1
MP:0010666	abnormal vitelline vein morphology	1
MP:0014004	absent basilar artery segment	1
MP:0008129	absent brain internal capsule	1
MP:0013998	absent canalicular internal carotid artery segment	1
MP:0008460	absent dorsal root ganglion	1
MP:0013880	absent ductus venosus	1
MP:0013914	absent intracranial segment of vertebral artery	1
MP:0013937	absent lobe of thyroid gland	1
MP:0000629	absent mammary gland	1
MP:0013926	absent neurohypophysis	1
MP:0013988	absent portal vein segment	1
MP:0013850	absent posterior commissure	1
MP:0000614	absent salivary gland	1
MP:0013823	absent segment of anterior cerebral artery	1
MP:0000690	absent spleen	1
MP:0008386	absent styloid process	1
MP:0002728	absent tibia	1
MP:0009905	absent tongue	1
MP:0001064	absent trochlear nerve	1
MP:0013595	absent vomeronasal organ	1
MP:0013860	anastomosis between common carotid and vertebral artery	1
MP:0014009	anastomosis between middle cerebral arteries	1
MP:0001293	anophthalmia	1
MP:0003387	aorta coarctation	1
MP:0006135	artery stenosis	1
MP:0000705	athymia	1
MP:0010403	atrial septal defect	1
MP:0013935	basal brain tissue herniation	1
MP:0010527	bicuspid pulmonary valve	1
MP:0011797	blind ureter	1
MP:0010607	common atrioventricular valve	1
MP:0004686	decreased length of long bones	1
MP:0009532	decreased parotid gland size	1
MP:0004648	decreased thoracic vertebrae number	1
MP:0011965	decreased total retina thickness	1
MP:0001247	dermal cysts	1
MP:0000825	dilated lateral ventricles	1
MP:0009144	dilated pancreatic duct	1
MP:0004938	dilated vasculature	1
MP:0011380	enlarged brain ventricles	1
MP:0013864	enlarged paraumbilical vein	1
MP:0003595	epididymal cyst	1
MP:0002947	increased hemangioma incidence	1
MP:0001634	internal hemorrhage	1
MP:0011974	intestinal stenosis	1
MP:0001916	intracerebral hemorrhage	1
MP:0003178	left pulmonary isomerism	1
MP:0013953	left sided brachiocephalic trunk	1
MP:0003327	liver cysts	1
MP:0003888	liver hemorrhage	1
MP:0000162	lordosis	1
MP:0010854	lung situs inversus	1
MP:0005287	narrow eye opening	1
MP:0004442	occipital bone foramen	1
MP:0000565	oligodactyly	1
MP:0006221	optic nerve hypoplasia	1
MP:0013933	short Meckel's cartilage	1
MP:0002766	situs inversus	1
MP:0002768	small adrenal glands	1
MP:0001306	small lens	1
MP:0013923	small prevertebral sympathetic ganglia	1
MP:0006254	thin cerebral cortex	1
MP:0013829	thin splanchnic nerve	1
MP:0013832	thin vagus nerve	1
MP:0003499	thyroid hypoplasia	1
MP:0009904	tongue hypoplasia	1
MP:0011697	vacuolated lens	1
MP:0013831	vagus nerve compression	1
MP:0004609	vertebral fusion	1

### Incidence of structural abnormalities in wild type embryos

To establish the possible impact of “background” abnormalities present within embryos irrespective of mutation, we also analysed a total of 114 wild type embryos, obtained from 41 of the 42 mutant lines ( Table 1). Previous large-scale studies of wild type E14.5 embryos from the same genetic background have enabled us to distinguish normal variation in structure from definite abnormalities, using careful stage-specific comparisons combined with statistical and morphometric analysis ^8^. This formed the basis for identifying phenotypes in the wild type embryos ( Table 2B and Supplementary Table 1B and Supplementary Table 5).

**Table 2B.** Frequency of phenotypes identified in wild type embryos. The Mammalian Phenotype Ontology terms describing phenotypes observed in each embryo were normalised to remove duplicates and the list then ranked in descending order by frequency of embryos exhibiting each phenotype.

MP ID	MP term	Frequency
MP:0002169	no abnormal phenotype detected	78
MP:0013971	blood in lymph vessels	5
MP:0011493	double ureter	4
MP:0013852	abnormal Mullerian duct topology	3
MP:0000783	abnormal forebrain morphology	3
MP:0013876	absent ductus venosus valve	3
MP:0013840	absent segment of posterior cerebral artery	3
MP:0011803	double kidney pelvis	3
MP:0003826	abnormal Mullerian duct morphology	2
MP:0013877	abnormal ductus venosus valve morphology	2
MP:0006063	abnormal inferior vena cava morphology	2
MP:0014003	additional anastomosis between intracranial vertebral arteries	2
MP:0003586	dilated ureter	2
MP:0011683	dual inferior vena cava	2
MP:0014021	heterochrony	2
MP:0013851	abnormal Wolffian duct topology	1
MP:0010595	abnormal aortic valve cusp morphology	1
MP:0002092	abnormal eye morphology	1
MP:0003686	abnormal eye muscle morphology	1
MP:0000559	abnormal femur morphology	1
MP:0013853	abnormal hepatic portal vein formation	1
MP:0000703	abnormal thymus morphology	1
MP:0013970	absent connection between subcutaneous lymph vessels and lymph sac	1
MP:0013835	absent hypoglossal nerve	1
MP:0000520	absent kidney	1
MP:0014006	absent posterior communicating artery	1
MP:0003722	absent ureter	1
MP:0006093	arteriovenous malformation	1
MP:0010530	cerebral arteriovenous malformation	1
MP:0013813	dilated hepatic portal vein	1
MP:0000602	enlarged liver sinusoidal spaces	1
MP:0002989	small kidney	1

In total, 56 phenotype calls were made, affecting 32 of the wild type embryos and 28 of the 41 lines. 21 of the 56 phenotype calls (38%) are accounted for by only 6 embryos, (indicating the skewing effect of a small number of abnormal embryos). Most affected embryos showing only a single phenotype. This is in marked contrast to the finding of many different phenotypes in individual mutant embryos.

The phenotypes of wild types vary in character, ranging from apparently minor differences (e.g. in blood vessel morphology) to a few major abnormalities (e.g. absent kidney). Each one is rare amongst the population of wild type embryos analysed and affects only a single wild type embryo within the line. Only 10 phenotypes (15 phenotype calls) overlap between mutant embryos and their wild type siblings and these affect only 10 of the 41 lines for which wild type embryos have been assessed ( Table 3).

**Table 3.** Overlap of identified phenotypes between homozygous mutant and wild type embryos within each line. Mutant lines showing a phenotype shared by at least one homozygous mutant and one wild type embryo are listed, along with the MP term, its MP ID and it penetrance amongst the mutant and wildtype embryos. For each line where an overlap is identified, the ratio of shared phenotypes to the total number of unique phenotypes identified in mutant embryos is also presented.

Allele	Phenotypes shared by homozygous mutants and wild type embryos	MP ID	Penetrance in mutants	Penetrance in wild types	Ratio of shared: total mutant phenotypes
Adamts3<tm1b(KOMP)Wtsi>	abnormal forebrain morphology	MP:0000783	2/7	1/3	2/44
	abnormal Mullerian duct topology	MP:0013852	1/7	1/3	
Adcy9<tm1b(EUCOMM)Wtsi>	abnormal Mullerian duct morphology	MP:0003826	1/8	1/3	2/20
	blood in lymph vessels	MP:0013971	1/8	1/3	
Celf4<tm1a(EUCOMM)Wtsi>	blood in lymph vessels	MP:0013971	1/5	1/3	1/7
Chtop<tm1a(EUCOMM)Wtsi>	abnormal forebrain morphology	MP:0000783	4/4	1/3	1/95
Cir1<tm3a(KOMP)Wtsi>	additional anastomosis between intracranial vertebral arteries	MP:0014003	1/3	1/2	1/29
Nsun2<tm1a(EUCOMM)Wtsi>	absent ductus venosus valve	MP:0013876	1/6	1/2	1/37
Psph<tm1a(EUCOMM)Hmgu>	blood in lymph vessels	MP:0013971	1/8	1/3	1/109
Tcf7l2<tm1a(EUCOMM)Wtsi>	absent ductus venosus valve	MP:0013876	2/5	1/4	3/32
	enlarged liver sinusoidal spaces	MP:0000602	2/5	1/4	
	abnormal eye muscle morphology	MP:0003686	3/5	1/4	
Traf6<tm2a(EUCOMM)Wtsi>	blood in lymph vessels	MP:0013971	4/9	1/5	1/39
Unk<tm1a(KOMP)Wtsi>	absent ureter	MP:0003722	2/5	1/2	2/10
	absent kidney	MP:0000520	2/5	1/2	

### Prevalence of individual abnormalities in mutant embryos

**Figure 2.**
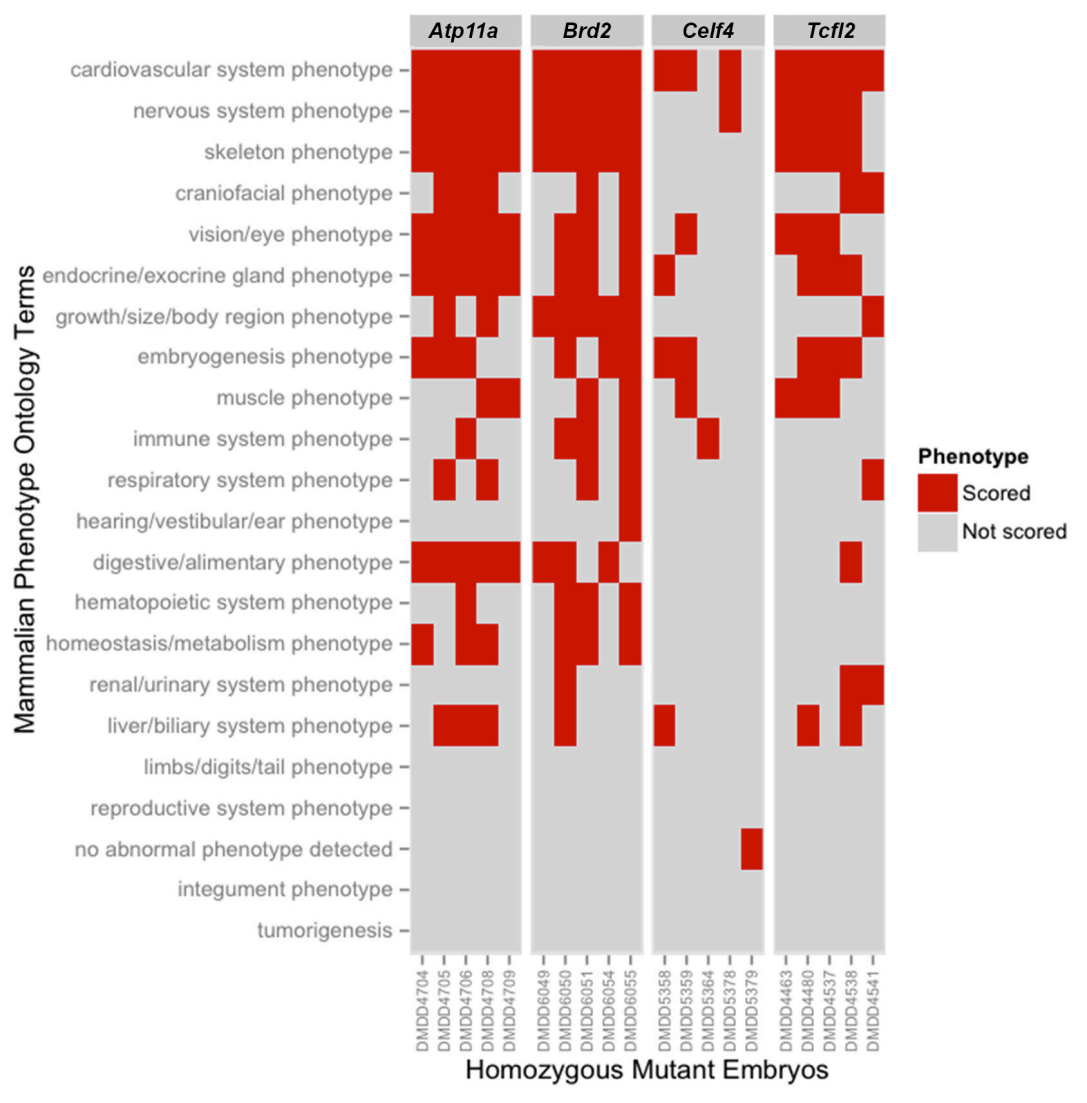
Individual mutant embryos show overlapping but distinct spectra of phenotypes. The phenotypes annotated for individual embryos were normalised to remove duplicate ontology terms. The distinct terms for each homozygous mutant embryo from four lines were then mapped onto the broad set of ontology categories defined in the high level DMDD slim. The presence or absence of phenotype annotation within each of the high level categories was plotted for each embryo analysed.

**Figure 3.**
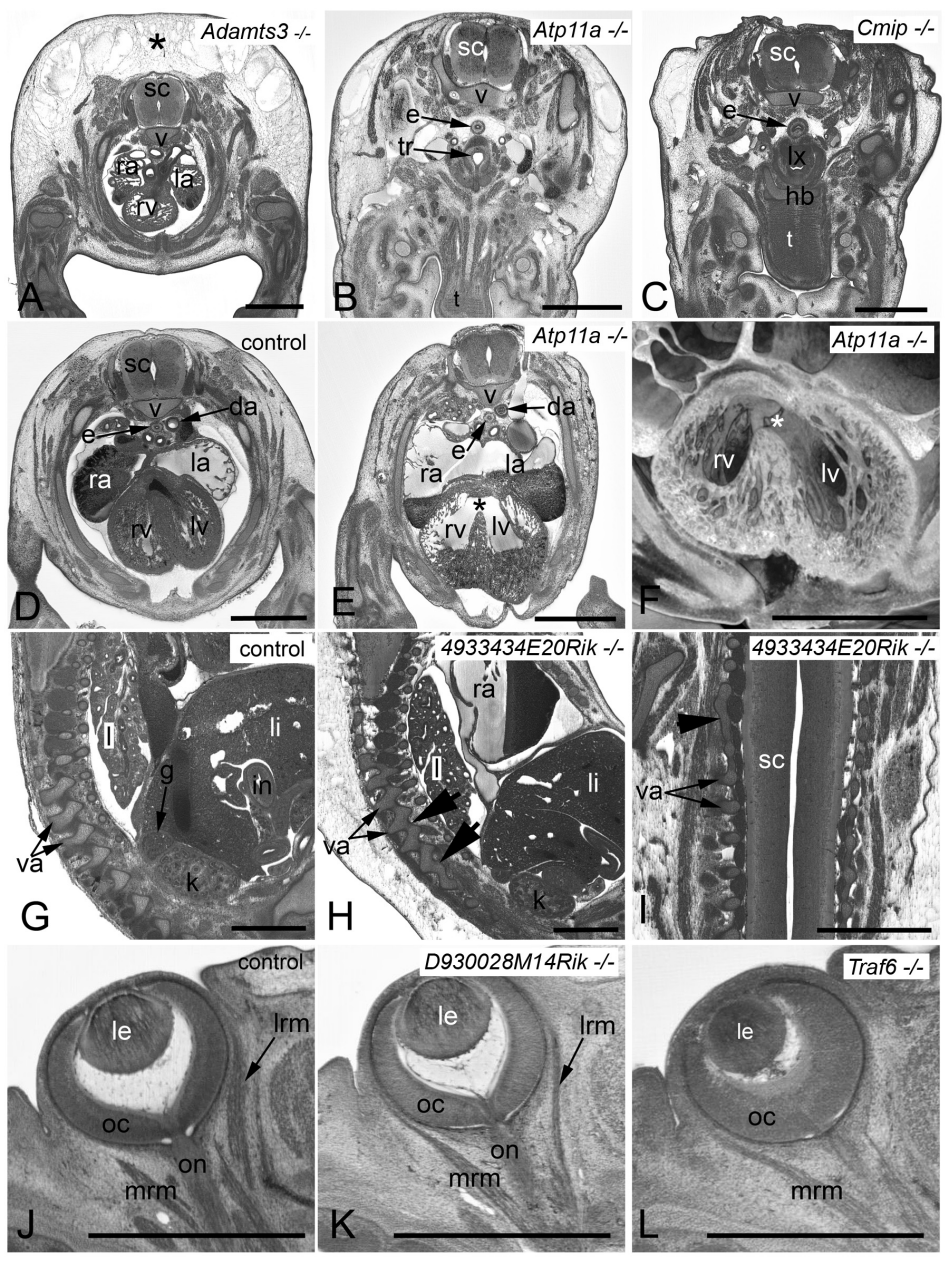
Examples of frequently observed abnormalities in mutant embryos. **A**– **C**. Subcutaneous edema. Original HREM sections showing a massive (asterisk) ( **A**), mild ( **B**), and unilaterally located subcutaneous edema ( **C**). Note the shrinkage artefacts in **B** and **C**, which complicate post mortem diagnosis. **D**– **F**. Perimembraneous septal defect. Normal situation in a control ( **D**) as appearing in an original HREM section. Defect (asterisk) as appearing in an original HREM section ( **E**) and a 3D volume model ( **F**). **G**– **I**. Fusion of vertebral arches. Normal situation in a control ( **G**) as appearing in a sagittal section. Fused articular processes (arrowheads) of subsequent vertebrae in a sagittal ( **H**) and a coronal section ( **I**). **J**– **L**. Abnormal eye muscle morphology as appearing in original HREM sections. Normal situation in a control ( **J**). Thinning of the lateral rectus muscle (lrm) ( **K**). Absence of the lateral rectus muscle (lrm) ( **L**). da, descending aorta; e, esophagus; g, adrenal gland; hb, hyoid bone; i, intestine; k, kidney; l, lung; la, left atrium; le, lens; li, liver; lrm, lateral rectus muscle; lv, left ventricle; lx, larynx; mrm, medial rectus muscle; oc, optic cup; on, optic nerve; ra, right atrium; rv, right ventricle; sc, spinal chord; t, tongue; tr, trachea; v, body of vertebra; va, arch of vertebra. Scale bars: 1 mm.

**Figure 4.**
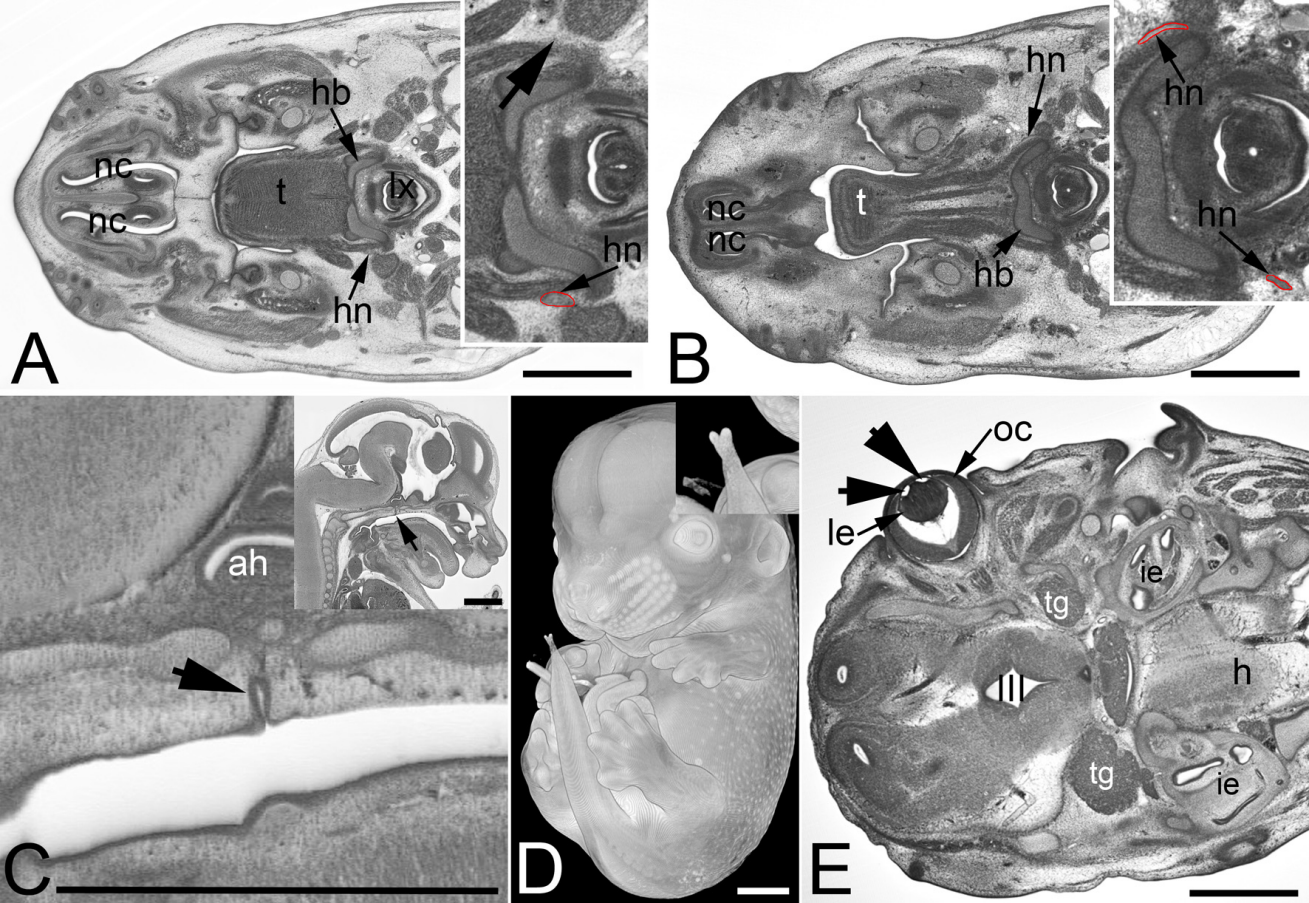
Other frequently observed abnormalities in mutant embryos. **A** and **B**. Abnormal hyopglossal nerve in original HREM sections through the head of *Prrc2b ^-/-^* ( **A**) and a *Polb ^-/-^* ( **B**) embryo. Note the missing right hypoglossal nerve (arrowhead, inlay) in **A** and the thinning of both hypoglossal nerves (hn) in **B**. **C**– **E**. Abnormalities that also occur in controls. Persisting craniopharnygeal duct (arrowhead) as appearing in sagittal sections ( **C**). Split tip of tail featured by volume models ( **D**) and vesicles (arrowheads) in the lens (le) as appearing in an original HREM section ( **E**).

**Figure 5.**
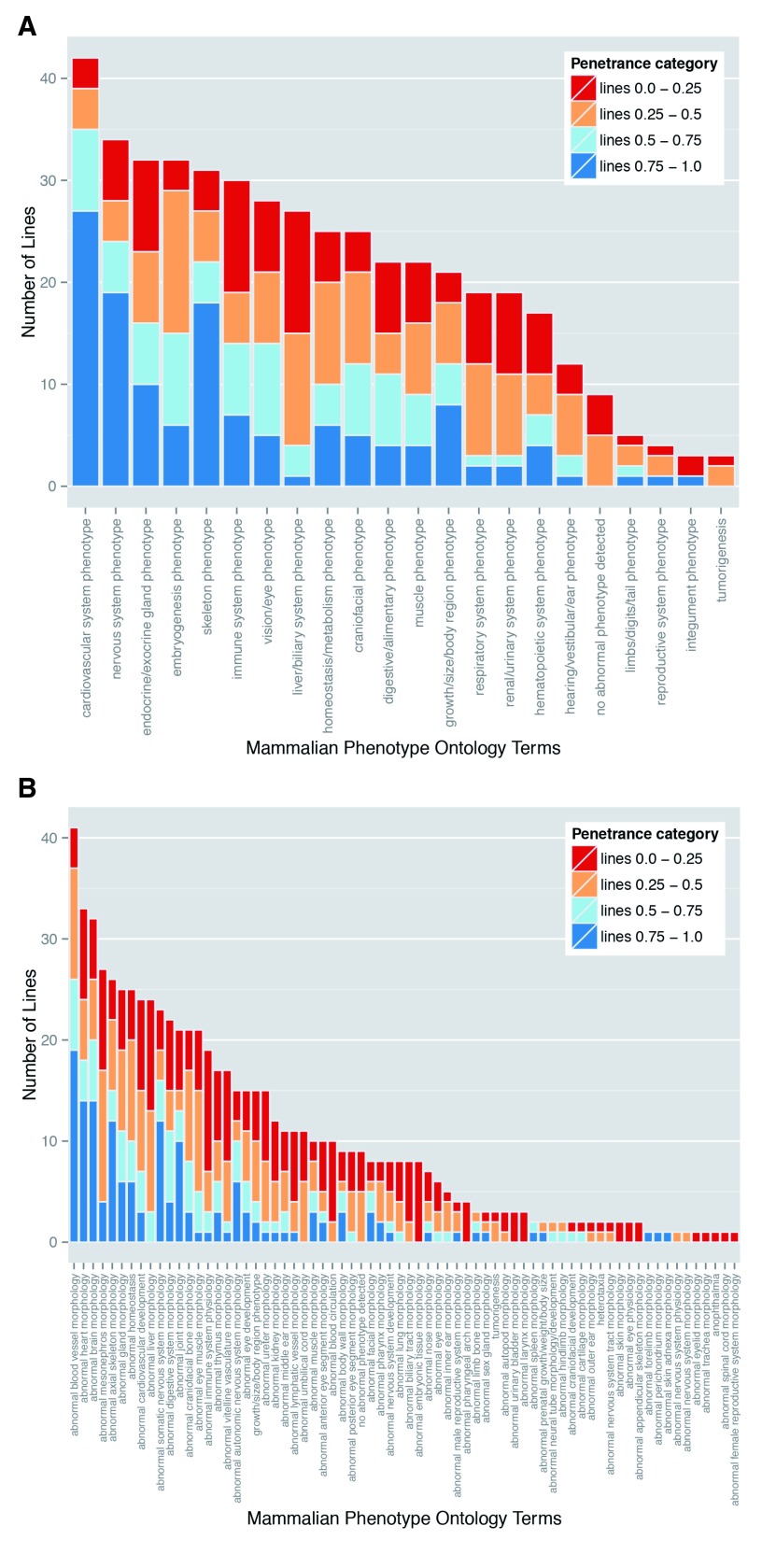
Variable prevalence and penetrance of individual phenotypes in mutant embryos. Data from the global analysis of the frequency of phenotype terms (see Materials and Methods) was plotted to show the number of lines falling into each of the observed phenotype categories. The colours indicate the number of lines falling into each of the distinct penetrance categories. The data was ordered according to line frequency, and subsequently by the numbers seen in the penetrance categories. ( **A**) shows the phenotype annotations summarised using the high level DMDD ontology slim, ( **B**) shows the phenotype annotations summarised using the intermediate level DMDD ontology slim.

Supplementary Table 1A presents the frequency of individual abnormalities that were identified amongst the mutant embryos. Since some phenotypes (such as vertebral abnormalities) are often present multiply in affected embryos, the data is normalised for occurrence by embryo. Interestingly, the most common phenotype detected in this study was subcutaneous edema. This was evident from macroscopic observation of embryos at harvest and confirmed by subsequent HREM imaging ( Figure 3, panels A–C). In total, subcutaneous edema and edema in other body regions (scored with four distinct MP terms) affected one third (72/220) of the embryos and was observed in a little over half (24/42) of the mutant lines. Other prevalent phenotypes included defects affecting the vertebral arches, the ventricular septum of the heart, forebrain morphology and musculature of the developing eyes ( Table 2A and Figure 3). Of particular note is the frequency with which mutant embryos showed abnormalities affecting the architecture or presence of the hypoglossal nerve ( Figure 4, panels A and B). Complete absence of the nerve occurred in 37 embryos, obtained from 12 different mutant lines, with some embryos from a similar number of lines showing abnormal topology or unusual thinness of the nerve (13 and 9 lines respectively). Overall, scored phenotypes affected all the major organ systems at E14.5 ( Figure 5A) and multiple organs or tissues were frequently affected within individual embryos, or collectively within a mutant line ( Figure 2 and Supplementary Figure 2 and Supplementary Figure 3). The complete listing of scored phenotypes is presented in Supplementary Table 4, organised according to the MP ontology slims adopted by the DMDD, with data ranked according to prevalence in mutant lines.

### Individual phenotypes show highly variable penetrance

Perhaps the most striking finding of the DMDD study is the almost complete absence of any fully penetrant abnormalities. Amongst lines for which more than a single embryo was analysed, only three phenotypes showed 100% penetrance: abnormal perichondrial ossification (1 line; 10 mutant embryos), small nodose ganglion (1 line; 4 embryos) and small trigeminal ganglion (1 line, 3 embryos). Furthermore, most defects showed surprisingly low penetrance. A penetrance greater than 75% within the line was only found for 7% of detected phenotypes. In contrast, over half (55%) of the scored abnormalities had a penetrance of 25% or less ( Table 4). This is graphically illustrated in Figure 5A, in which the scored phenotypes are clustered according to high level MP ontology terms (broadly reflecting distinct organ systems, tissues or body regions) and the prevalence of each in the 42 mutant lines categorised by penetrance. All phenotypes show a broad range of penetrance, about half showing roughly symmetrical distribution of penetrance, with similar numbers of lines both above and below 50%. Interestingly, it is possible also to distinguish several phenotypes where penetrance is noticeably skewed. Abnormalities affecting the cardiovascular system, nervous system and skeleton all affected a relatively large number of lines and each showed a striking bias towards higher penetrance values. A second group of abnormalities encompassing liver/biliary, respiratory, renal and hearing systems showed a converse bias to penetrance values below 50% ( Figure 5A).

**Table 4.** Variability in mutant phenotype penetrance. Every distinct phenotype scored in each line was listed along with its penetrance (i.e. the number of embryos showing the phenotype divided by the total number of embryos analysed for that line). Scored phenotypes were then ranked by penetrance value to obtain the proportions falling within the four ranges shown. (Note that all data from the lines *Otud7b*, *Npat* and *Dhx35* were removed from the analysis, since in each case, these were obtained from examination of a single embryo).

Penetrance range	Phenotypes scored (homozygous mutants)	%
<25%	673	55.21%
26–50%	343	28.14%
51–75%	118	9.68%
>75%	85	6.97%

**Table 5.** High level MP ontology slim used by DMDD. A list of the Mammalian Phenotype Ontology IDs and names of terms selected as the high level ontology slim.

MP:0002169	no abnormal phenotype detected
MP:0005375	adipose tissue phenotype
MP:0005386	behavior/neurological phenotype
MP:0005385	cardiovascular system phenotype
MP:0005384	cellular phenotype
MP:0005382	craniofacial phenotype
MP:0005381	digestive/alimentary phenotype
MP:0005380	embryogenesis phenotype
MP:0005379	endocrine/exocrine gland phenotype
MP:0005378	growth/size/body region phenotype
MP:0005377	hearing/vestibular/ear phenotype
MP:0005397	hematopoietic system phenotype
MP:0005376	homeostasis/metabolism phenotype
MP:0005387	immune system phenotype
MP:0010771	integument phenotype
MP:0005371	limbs/digits/tail phenotype
MP:0005370	liver/biliary system phenotype
MP:0010768	mortality/aging
MP:0005369	muscle phenotype
MP:0003631	nervous system phenotype
MP:0001186	pigmentation phenotype
MP:0005367	renal/urinary system phenotype
MP:0005389	reproductive system phenotype
MP:0005388	respiratory system phenotype
MP:0005390	skeleton phenotype
MP:0005394	taste/olfaction phenotype
MP:0002006	tumorigenesis
MP:0005391	vision/eye phenotype

**Table 6.** Intermediate level MP ontology slim used by DMDD. A list of the Mammalian Phenotype Ontology IDs and names of terms selected as the intermediate level ontology slim.

MP:0000001	mammalian phenotype
MP:0002873	normal phenotype
MP:0002169	no abnormal phenotype detected
MP:0005375	adipose tissue phenotype
MP:0000003	abnormal adipose tissue morphology
MP:0005666	abnormal adipose tissue physiology
MP:0004924	abnormal behavior
MP:0020222	abnormal alertness
MP:0011275	abnormal behavioral response to light
MP:0009745	abnormal behavioral response to xenobiotic
MP:0001502	abnormal circadian rhythm
MP:0002069	abnormal consumption behavior
MP:0002572	abnormal emotion/affect behavior
MP:0001440	abnormal grooming behavior
MP:0010698	abnormal impulsive behavior control
MP:0002063	abnormal learning/memory/conditioning
MP:0002066	abnormal motor capabilities/coordination/ movement
MP:0002067	abnormal sensory capabilities/reflexes/ nociception
MP:0011396	abnormal sleep behavior
MP:0002557	abnormal social/conspecific interaction
MP:0001529	abnormal vocalization
MP:0002822	catalepsy
MP:0002899	fatigue
MP:0002064	seizures
MP:0002127	abnormal cardiovascular system morphology
MP:0001614	abnormal blood vessel morphology
MP:0002925	abnormal cardiovascular development
MP:0000266	abnormal heart morphology
MP:0003279	aneurysm
MP:0013332	peliosis
MP:0001544	abnormal cardiovascular system physiology
MP:0002128	abnormal blood circulation
MP:0010695	abnormal blood pressure regulation
MP:0000249	abnormal blood vessel physiology
MP:0004039	abnormal cardiac cell glucose uptake
MP:0002972	abnormal cardiac muscle contractility
MP:0004084	abnormal cardiac muscle relaxation
MP:0011926	abnormal cardiac valve physiology
MP:0011390	abnormal fetal cardiomyocyte physiology
MP:0011925	abnormal heart echocardiography feature
MP:0008775	abnormal heart ventricle pressure
MP:0004085	abnormal heartbeat
MP:0003137	abnormal impulse conducting system conduction
MP:0020095	abnormal mean heart rate adaptation
MP:0004215	abnormal myocardial fiber physiology
MP:0003547	abnormal pulmonary pressure
MP:0020092	abnormal susceptibility to aortic cartilaginous metaplasia
MP:0020098	abnormal susceptibility to diet-induced aortic fatty streak lesions
MP:0000230	abnormal systemic arterial blood pressure
MP:0004484	altered response of heart to induced stress
MP:0000343	altered response to myocardial infarction
MP:0005330	cardiomyopathy
MP:0006138	congestive heart failure
MP:0001853	heart inflammation
MP:0003328	portal hypertension
MP:0005384	cellular phenotype
MP:0000358	abnormal cell morphology
MP:0005621	abnormal cell physiology
MP:0013258	abnormal extracellular matrix morphology
MP:0003121	genetic imprinting
MP:0005382	craniofacial phenotype
MP:0000428	abnormal craniofacial morphology
MP:0002116	abnormal craniofacial bone morphology
MP:0003935	abnormal craniofacial development
MP:0003743	abnormal facial morphology
MP:0011495	abnormal head shape
MP:0002177	abnormal outer ear morphology
MP:0005381	digestive/alimentary phenotype
MP:0000462	abnormal digestive system morphology
MP:0001663	abnormal digestive system physiology
MP:0005380	embryogenesis phenotype
MP:0001672	abnormal embryogenesis/development
MP:0002084	abnormal developmental patterning
MP:0001697	abnormal embryo size
MP:0002085	abnormal embryonic tissue morphology
MP:0008926	abnormal anterior definitive endoderm morphology
MP:0013230	abnormal cervical sinus morphology
MP:0003085	abnormal egg cylinder morphology
MP:0010115	abnormal embryonic cloaca morphology
MP:3000001	abnormal gastrula morphology
MP:0011411	abnormal gonadal ridge morphology
MP:0011257	abnormal head fold morphology
MP:0011260	abnormal head mesenchyme morphology
MP:0012187	abnormal intraembryonic coelom morphology
MP:0005650	abnormal limb bud morphology
MP:0006301	abnormal mesenchyme morphology
MP:0008487	abnormal mesonephros morphology
MP:0011256	abnormal neural fold morphology
MP:0005657	abnormal neural plate morphology
MP:0002151	abnormal neural tube morphology/ development
MP:0002825	abnormal notochord morphology
MP:0002884	abnormal pharyngeal arch morphology
MP:0013231	abnormal pharyngeal groove morphology
MP:0013232	abnormal pharyngeal membrane morphology
MP:0006031	abnormal pharyngeal pouch morphology
MP:0012496	abnormal pleuropericardial membrane morphology
MP:0002399	abnormal pluripotent precursor cell morphology/development
MP:0013217	abnormal posterior definitive endoderm morphology
MP:0003885	abnormal rostral-caudal body axis extension
MP:0012252	abnormal septum transversum morphology
MP:0001688	abnormal somite development
MP:0002861	abnormal tail bud morphology
MP:0011258	abnormal tail fold morphology
MP:0001674	abnormal triploblastic development
MP:0011835	abnormal urogenital fold morphology
MP:0011853	abnormal urorectal septum morphology
MP:0003988	disorganized embryonic tissue
MP:0013241	embryo tissue necrosis
MP:0008932	abnormal embryonic tissue physiology
MP:0003890	abnormal embryonic-extraembryonic boundary morphology
MP:0002086	abnormal extraembryonic tissue morphology
MP:0001726	abnormal allantois morphology
MP:0005029	abnormal amnion morphology
MP:0011199	abnormal amniotic cavity morphology
MP:0002836	abnormal chorion morphology
MP:0011202	abnormal ectoplacental cavity morphology
MP:0003396	abnormal embryonic hematopoiesis
MP:0011200	abnormal extraembryonic coelom morphology
MP:0010736	abnormal extraembryonic ectoderm morphology
MP:0001724	abnormal extraembryonic endoderm formation
MP:0006323	abnormal extraembryonic mesoderm development
MP:0011203	abnormal parietal yolk sac morphology
MP:0001711	abnormal placenta morphology
MP:0011197	abnormal proamniotic cavity morphology
MP:0001725	abnormal umbilical cord morphology
MP:0011201	abnormal visceral yolk sac cavity morphology
MP:0001718	abnormal visceral yolk sac morphology
MP:0003229	abnormal vitelline vasculature morphology
MP:0002582	disorganized extraembryonic tissue
MP:0004264	abnormal extraembryonic tissue physiology
MP:0004966	abnormal inner cell mass proliferation
MP:0009781	abnormal preimplantation embryo development
MP:0011186	abnormal visceral endoderm morphology
MP:0012028	abnormal visceral endoderm physiology
MP:0001730	embryonic growth arrest
MP:0003984	embryonic growth retardation
MP:0005379	endocrine/exocrine gland phenotype
MP:0002163	abnormal gland morphology
MP:0002164	abnormal gland physiology
MP:0005378	growth/size/body region phenotype
MP:0009701	abnormal birth body size
MP:0005451	abnormal body composition
MP:0003385	abnormal body wall morphology
MP:0004134	abnormal chest morphology
MP:0000432	abnormal head morphology
MP:0012719	abnormal neck morphology
MP:0002089	abnormal postnatal growth/weight/body size
MP:0004196	abnormal prenatal growth/weight/body size
MP:0001270	distended abdomen
MP:0004133	heterotaxia
MP:0013328	visceromegaly
MP:0005377	hearing/vestibular/ear phenotype
MP:0002102	abnormal ear morphology
MP:0003938	abnormal ear development
MP:0000026	abnormal inner ear morphology
MP:0000049	abnormal middle ear morphology
MP:0002177	abnormal outer ear morphology
MP:0003878	abnormal ear physiology
MP:0005397	hematopoietic system phenotype
MP:0002396	abnormal hematopoietic system morphology/ development
MP:0002429	abnormal blood cell morphology/development
MP:0002398	abnormal bone marrow cell morphology/ development
MP:0004808	abnormal hematopoietic stem cell morphology
MP:0000689	abnormal spleen morphology
MP:0000703	abnormal thymus morphology
MP:0001545	abnormal hematopoietic system physiology
MP:0005376	homeostasis/metabolism phenotype
MP:0001764	abnormal homeostasis
MP:0005266	abnormal metabolism
MP:0008872	abnormal physiological response to xenobiotic
MP:0005164	abnormal response to injury
MP:0000604	amyloidosis
MP:0013027	wounding
MP:0005387	immune system phenotype
MP:0000685	abnormal immune system morphology
MP:0000716	abnormal immune system cell morphology
MP:0002722	abnormal immune system organ morphology
MP:0001879	abnormal lymphatic vessel morphology
MP:0001790	abnormal immune system physiology
MP:0010771	integument phenotype
MP:0010678	abnormal skin adnexa morphology
MP:0010680	abnormal skin adnexa physiology
MP:0002060	abnormal skin morphology
MP:0005501	abnormal skin physiology
MP:0001968	abnormal touch/nociception
MP:0005371	limbs/digits/tail phenotype
MP:0002109	abnormal limb morphology
MP:0000572	abnormal autopod morphology
MP:0000550	abnormal forelimb morphology
MP:0000556	abnormal hindlimb morphology
MP:0002115	abnormal limb bone morphology
MP:0006279	abnormal limb development
MP:0012000	abnormal limb position
MP:0000549	absent limbs
MP:0008985	hemimelia
MP:0013069	limb wound
MP:0000548	long limbs
MP:0013133	pale limbs
MP:0000547	short limbs
MP:0020288	supernumerary limbs
MP:0002111	abnormal tail morphology
MP:0005370	liver/biliary system phenotype
MP:0002138	abnormal hepatobiliary system morphology
MP:0005083	abnormal biliary tract morphology
MP:0003943	abnormal hepatobiliary system development
MP:0000598	abnormal liver morphology
MP:0010040	abnormal oval cell morphology
MP:0002139	abnormal hepatobiliary system physiology
MP:0010768	mortality/aging
MP:0005369	muscle phenotype
MP:0002108	abnormal muscle morphology
MP:0002106	abnormal muscle physiology
MP:0003631	nervous system phenotype
MP:0003632	abnormal nervous system morphology
MP:0002751	abnormal autonomic nervous system morphology
MP:0002152	abnormal brain morphology
MP:0002653	abnormal ependyma morphology
MP:0003634	abnormal glial cell morphology
MP:0002184	abnormal innervation
MP:0005623	abnormal meninges morphology
MP:0003861	abnormal nervous system development
MP:0000778	abnormal nervous system tract morphology
MP:0002882	abnormal neuron morphology
MP:0002752	abnormal somatic nervous system morphology
MP:0000955	abnormal spinal cord morphology
MP:0008493	alpha-synuclein inclusion body
MP:0003329	amyloid beta deposits
MP:0012260	encephalomeningocele
MP:0002229	neurodegeneration
MP:0003012	no phenotypic analysis
MP:0005395	other phenotype
MP:0001186	pigmentation phenotype
MP:0005367	renal/urinary system phenotype
MP:0000516	abnormal renal/urinary system morphology
MP:0011782	abnormal internal urethral orifice morphology
MP:0002135	abnormal kidney morphology
MP:0005187	abnormal penis morphology
MP:0000534	abnormal ureter morphology
MP:0011487	abnormal ureteropelvic junction morphology
MP:0011488	abnormal ureterovesical junction morphology
MP:0000537	abnormal urethra morphology
MP:0000538	abnormal urinary bladder morphology
MP:0003942	abnormal urinary system development
MP:0003630	abnormal urothelium morphology
MP:0003129	persistent cloaca
MP:0005360	urolithiasis
MP:0005502	abnormal renal/urinary system physiology
MP:0003633	abnormal nervous system physiology
MP:0005389	reproductive system phenotype
MP:0002160	abnormal reproductive system morphology
MP:0001119	abnormal female reproductive system morphology
MP:0001929	abnormal gametogenesis
MP:0005149	abnormal gubernaculum morphology
MP:0003673	abnormal inguinal canal morphology
MP:0001145	abnormal male reproductive system morphology
MP:0003315	abnormal perineum morphology
MP:0003936	abnormal reproductive system development
MP:0002210	abnormal sex determination
MP:0000653	abnormal sex gland morphology
MP:0013055	genital wound
MP:0001919	abnormal reproductive system physiology
MP:0005388	respiratory system phenotype
MP:0002132	abnormal respiratory system morphology
MP:0002249	abnormal larynx morphology
MP:0001175	abnormal lung morphology
MP:0002233	abnormal nose morphology
MP:0002240	abnormal paranasal sinus morphology
MP:0002234	abnormal pharynx morphology
MP:0010820	abnormal pleura morphology
MP:0012684	abnormal pleural cavity morphology
MP:0010942	abnormal respiratory epithelium morphology
MP:0003115	abnormal respiratory system development
MP:0002282	abnormal trachea morphology
MP:0002133	abnormal respiratory system physiology
MP:0005390	skeleton phenotype
MP:0005508	abnormal skeleton morphology
MP:0009250	abnormal appendicular skeleton morphology
MP:0002114	abnormal axial skeleton morphology
MP:0003795	abnormal bone structure
MP:0000163	abnormal cartilage morphology
MP:0011849	abnormal clitoral bone morphology
MP:0002932	abnormal joint morphology
MP:0005504	abnormal ligament morphology
MP:0006322	abnormal perichondrium morphology
MP:0002113	abnormal skeleton development
MP:0005503	abnormal tendon morphology
MP:0000566	synostosis
MP:0001533	abnormal skeleton physiology
MP:0005394	taste/olfaction phenotype
MP:0005500	abnormal gustatory system morphology
MP:0001002	abnormal taste bud morphology
MP:0001985	abnormal gustatory system physiology
MP:0005499	abnormal olfactory system morphology
MP:0006292	abnormal nasal placode morphology
MP:0008789	abnormal olfactory epithelium morphology
MP:0012067	abnormal olfactory gland morphology
MP:0001983	abnormal olfactory system physiology
MP:0002006	tumorigenesis
MP:0005391	vision/eye phenotype
MP:0002092	abnormal eye morphology
MP:0005193	abnormal anterior eye segment morphology
MP:0001286	abnormal eye development
MP:0001299	abnormal eye distance/position
MP:0003686	abnormal eye muscle morphology
MP:0001324	abnormal eye pigmentation
MP:0002697	abnormal eye size
MP:0001340	abnormal eyelid morphology
MP:0008968	abnormal lacrimal apparatus morphology
MP:0010030	abnormal orbit morphology
MP:0005195	abnormal posterior eye segment morphology
MP:0002698	abnormal sclera morphology
MP:0005197	abnormal uvea morphology
MP:0001293	anophthalmia
MP:0006209	calcified intraocular region
MP:0013146	eye lesions
MP:0009859	eye opacity
MP:0013170	eye swellings
MP:0006225	ocular rupture
MP:0001788	periorbital edema
MP:0005254	strabismus
MP:0005253	abnormal eye physiology

**Table 7.** New MP terms derived from embryo phenotyping. A list of the Mammalian Phenotype Ontology IDs along with their corresponding term name. These have been added to the ontology to allow annotation of abnormalities observed in the embryos which could not be adequately described by existing terms.

MP:0013809	absent pectinate muscle
	
MP:0013810	absent brachiocephalic trunk
MP:0013812	enlarged orbital veins
MP:0013813	dilated hepatic portal vein
MP:0013814	abnormal hepatic portal vein connection
MP:0013816	absent digastric muscle
MP:0013817	absent nasal cavity
MP:0013818	abnormal oral cavity morphology
MP:0013819	abnormal acromioclavicular joint morphology
MP:0013820	absent optic cup
MP:0013823	absent segment of anterior cerebral artery
MP:0013825	small hypoglossal canal
MP:0013826	absent hypoglossal canal
MP:0013827	thin oculomotor nerve
MP:0013828	thin facial nerve
MP:0013829	thin splanchnic nerve
MP:0013830	abnormal intrathoracic topology of vagus nerve
MP:0013831	vagus nerve compression
MP:0013832	thin vagus nerve
MP:0013833	absent olfactory nerve
MP:0013834	thin hypoglossal nerve
MP:0013835	absent hypoglossal nerve
MP:0013836	abnormal hypoglossal nerve topology
MP:0013837	abnormal vagus nerve topology
MP:0013838	small caudate nucleus
MP:0013840	absent segment of posterior cerebral artery
MP:0013841	abnormal lymphatic vessel topology
MP:0013842	ductus venosus stenosis
MP:0013843	hepatic portal vein stenosis
MP:0013844	abnormal perichondrial ossification
MP:0013845	abnormal eye muscle topology
MP:0013846	retropharyngeal edema
MP:0013847	retropleural edema
MP:0013848	subcutaneous edema
MP:0013849	absent abducens nerve
MP:0013850	absent posterior commissure
MP:0013851	abnormal Wolffian duct topology
MP:0013852	abnormal Mullerian duct topology
MP:0013853	abnormal hepatic portal vein formation
MP:0013855	absent celiac artery
MP:0013857	abnormal abdominal muscle morphology
MP:0013858	abnormal azygos vein topology
MP:0013859	abnormal vitelline vein connection
MP:0013860	anastomosis between common carotid and vertebral artery
MP:0013861	abnormal pancreas topology
MP:0013862	abnormal cecum position
MP:0013864	enlarged paraumbilical vein
MP:0013865	abnormal dorsal pancreas topology
MP:0013868	abnormal ventral pancreas topology
MP:0013869	vascular diverticulum
MP:0013870	absent proximal internal carotid artery segment
MP:0013871	abnormal stapedial artery topology
MP:0013873	abnormal ductus venosus morphology
MP:0013874	abnormal ductus venosus topology
MP:0013875	trigeminal neuroma
MP:0013876	absent ductus venosus valve
MP:0013877	abnormal ductus venosus valve morphology
MP:0013878	abnormal ductus venosus valve topology
MP:0013879	duplication of ductus venosus
MP:0013880	absent ductus venosus
MP:0013913	absent rib-vertebral column attachment
MP:0013914	absent intracranial segment of vertebral artery
MP:0013915	abnormal brachial plexus formation
MP:0013916	decreased intestine length
MP:0013917	persistent right 6th pharyngeal arch artery
MP:0013918	abnormal endolymphatic sac topology
MP:0013923	small prevertebral sympathetic ganglia
MP:0013924	abnormal dural venous sinus morphology
MP:0013925	abnormal vascular plexus formation
MP:0013926	absent neurohypophysis
MP:0013927	abnormal facial nerve topology
MP:0013928	thin motoric part of trigeminal nerve
MP:0013929	absent eye muscles
MP:0013930	abnormal digastric muscle connection
MP:0013931	abnormal olfactory bulb position
MP:0013932	fragmented Meckel's cartilage
MP:0013933	short Meckel's cartilage
MP:0013934	supratentorial ventricles enlargement
MP:0013935	basal brain tissue herniation
MP:0013936	abnormal thymus topology
MP:0013937	absent lobe of thyroid gland
MP:0013938	abnormal esophagus topology
MP:0013943	abnormal ureter topology
MP:0013944	persistent cloacal membrane
MP:0013945	abnormal elbow joint morphology
MP:0013946	abnormal perirectal tissue morphology
MP:0013947	abnormal paraaortic body morphology
MP:0013948	intraembryonal intestine elongation
MP:0013949	fusion of axis and occipital bones
MP:0013950	abnormal dorsal root ganglion topology
MP:0013951	abnormal descending aorta topology
MP:0013952	retro-esophageal left subclavian artery
MP:0013953	left sided brachiocephalic trunk
MP:0013963	jugular vein stenosis
MP:0013964	absent tongue muscles
MP:0013965	abnormally deep median sulcus of tongue
MP:0013967	abnormal infrahyoid muscle connection
MP:0013968	multiple persisting craniopharyngeal ducts
MP:0013969	reduced sympathetic cervical ganglion size
MP:0013970	absent connection between subcutaneous lymph vessels and lymph sac
MP:0013971	blood in lymph vessels
MP:0013972	occipital vertebra
MP:0013973	abnormal hepatic vein connection
MP:0013974	abnormal coronary vein connection
MP:0013975	abnormal coronary sinus connection
MP:0013976	abnormal left vena cava superior connection
MP:0013977	symmetric azygos veins
MP:0013978	abnormal carotid artery origin
MP:0013979	abnormal subclavian artery origin
MP:0013980	abnormal pulmonary artery origin
MP:0013981	double lumen aortic arch
MP:0013982	inverse situs of great intrathoracic arteries
MP:0013984	abnormal superior mesenterial vein connection
MP:0013985	abnormal umbilical vein topology
MP:0013986	abnormal vitelline vein topology
MP:0013987	absent intrahepatic inferior vena cava segment
MP:0013988	absent portal vein segment
MP:0013989	symmetric hepatic veins
MP:0013991	abnormal common iliac artery origin
MP:0013992	persistent dorsal ophthalmic artery
MP:0013993	anastomosis between basilar artery and common carotid artery
MP:0013994	abnormal parasellar internal carotid artery branch morphology
MP:0013995	abnormal external carotid artery origin
MP:0013996	abnormal vertebral artery origin
MP:0013997	abnormal internal carotid artery topology
MP:0013998	absent canalicular internal carotid artery segment
MP:0013999	absent parasellar internal carotid artery
MP:0014000	anastomosis between internal carotid artery and basilar artery
MP:0014001	abnormal vertebral artery topology
MP:0014002	absent extracranial vertebral artery segment
MP:0014003	additional anastomosis between intracranial vertebral arteries
MP:0014004	absent basilar artery segment
MP:0014006	absent posterior communicating artery
MP:0014008	absent labyrinthine artery
MP:0014009	anastomosis between middle cerebral arteries
MP:0014011	abnormal ovary tissue architecture
MP:0014017	abnormal Wolffian duct connection
MP:0014018	embryo tumor
MP:0014019	embryo cyst
MP:0014020	intramural bleeding in blood vessel wall
MP:0014021	heterochrony
MP:0014022	abnormal duodenum topology

**Figure 6.**
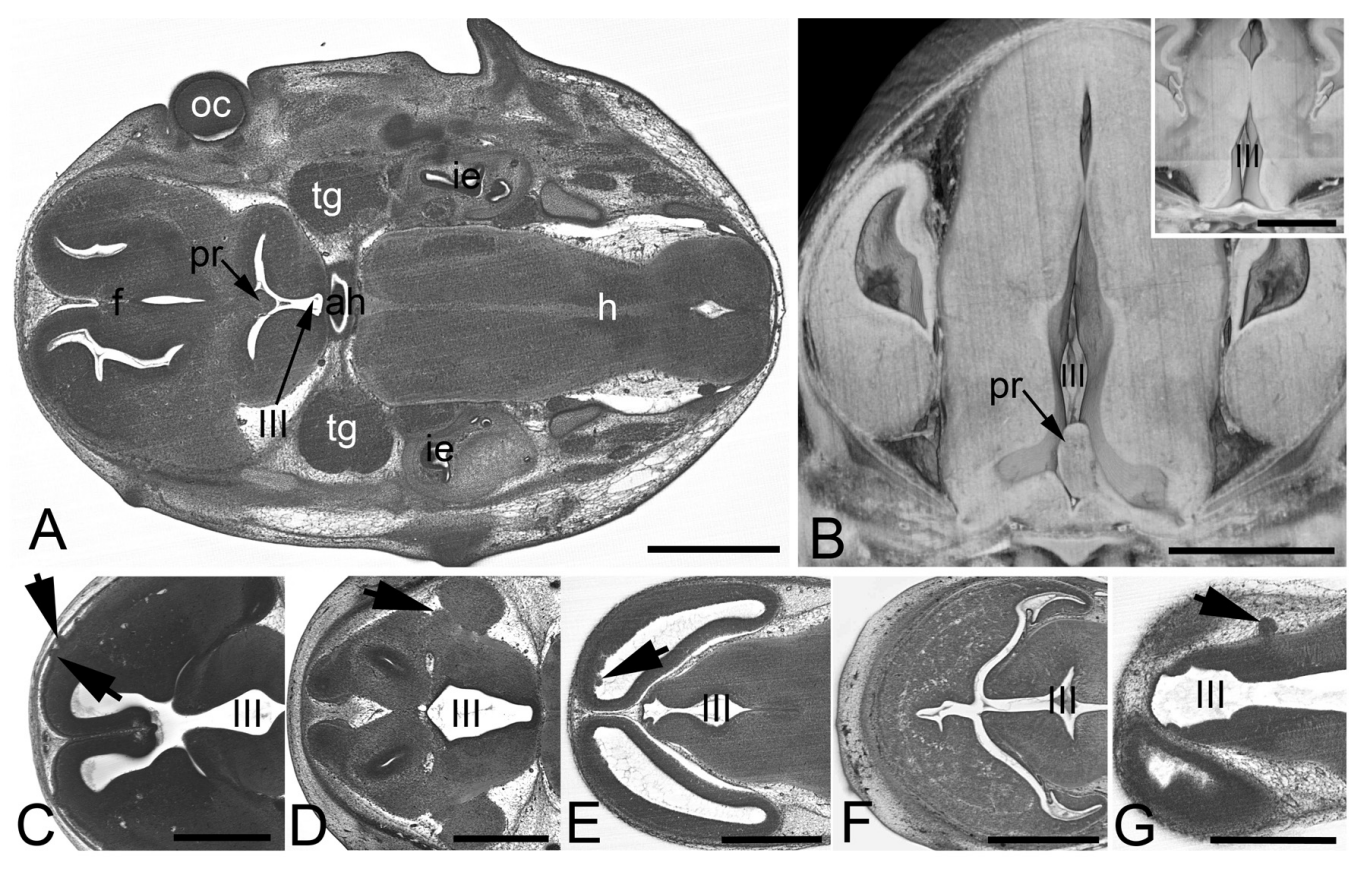
Abnormal brain morphology phenotypes. **A** and **B**. Tissue protrusion (pr) into the 3rd ventricle (III) in an original HREM-section ( **A**) and a volume model ( **B**). Inlay in **B** shows normal situation in a control. **C**. Irregular tissue protrusions (arrowheads) on the brain surface in a *4933434E20Rik ^-/-^* embryo. **D**. Abnormal tissue (arrowhead) at the cortex near the lateral sulcus in a *Polb ^-/-^* embryo. **E**. Abnormal frontal wall of the lateral ventricles in a *H13 ^-/-^* embryo. **F**. Abnormal morphology and tissue architecture (arrowhead) of the frontal forebrain in a *Chtop ^-/-^* embryo. **G**. Abnormal morphology of the wall of the 3rd ventricle and protrusions (arrowhead) on the surface of the diencephalon in a *Brd2 ^-/-^* embryo. ah, adenohypophysis; f, forebrain; h, hindbrain; ie, inner ear; oc, optic cup; pr, tissue protrusion; tg, trigeminal ganglion; III, 3rd ventricle; Scale bars 1 mm.

When grouped into such high level MP ontology terms, the most common group of abnormalities are those affecting the cardiovascular system, examples of which affect embryos in every single mutant line studied. Almost as prevalent are nervous system phenotypes, which are detected in 80% of the lines studied. Re-plotting the data summarised by intermediate level MP term slim provides a more detailed view of the prevalence and variability in penetrance of phenotypes ( Figure 5B). At this level of resolution, for example, cardiovascular defects are subdivided into two broad categories; those encompassing abnormalities in blood vessel morphology or topology (“abnormal blood vessel morphology” and most phenotypes within “abnormal cardiovascular development”) and those affecting the heart and its great vessels (“abnormal heart morphology”). Viewed in this way, it is clear that detection of cardiovascular defects in all lines examined results from the presence of phenotypes in the vasculature. These range from relatively major defects such as absence of the ductus venosus, interrupted aortic arch or arterial stenosis, to more minor alterations in vascular topology in different regions of the embryo. Cardiac abnormalities nevertheless remain prevalent, affecting almost two thirds (27/42) of the mutant lines. These encompass malformations in all regions of the four-chambered heart and its great vessels, including both atrial and ventricular septal defects, atrioventricular septal defects, common arterial trunk, double outlet right ventricle, transposition of the great arteries, bicuspid aortic valve, common truncal valve and abnormally thin myocardium. After blood vessel and cardiac abnormalities, the third most prevalent group of phenotypes detected were those affecting brain morphology ( Figure 5B), most commonly the forebrain ( Figure 6 and Supplementary Table 1A).

**Figure 7.**
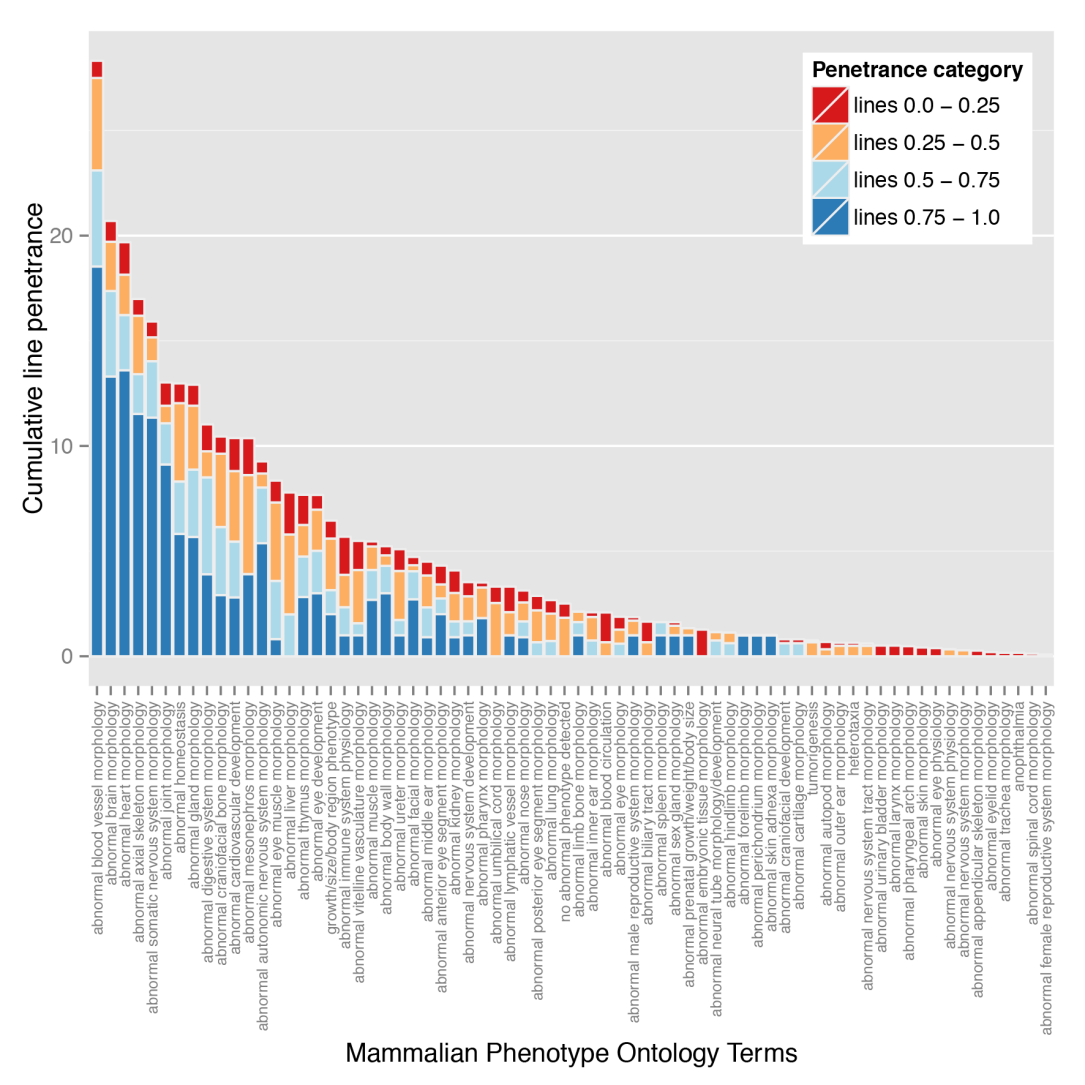
Cumulative penetrance of individual phenotypes in mutant embryos. Data from the global analysis of the frequency of phenotype terms (see Materials and Methods) was plotted to show the cumulative penetrance score for each of the phenotype categories observed (i.e. the overall sum of the penetrance scores recorded for the lines showing the phenotype). The Mammalian Phenotype Ontology terms assigned during embryo phenotyping were summarised using the intermediate level DMDD ontology slim, and the data was ordered according to the cumulative penetrance score. The colours indicate the contribution of lines falling into each of the distinct penetrance categories to the cumulative penetrance score.

In order to assess the relative significance of each phenotype in the context of variable penetrance, we re-examined their ranking distribution after weighting each phenotype according to its individual prevalence. This provides a plot of cumulative line penetrance for each of the 70 intermediate level MP term slim ( Figure 7). Whilst abnormalities in blood vessel morphology and structure of the heart remain amongst the most prevalent phenotypes, weighting by penetrance has a significant impact on the ranking of other phenotypes. Notably, the relative ranking of “abnormal brain morphology” and “abnormal somatic nervous system morphology” is increased, with both now lying in the five most prevalent abnormalities scored. This change is largely driven by the relatively high prevalence associated with abnormalities in forebrain morphology and hypoglossal nerve structure or presence, respectively.

### Phenotype penetrance is affected by allele type

**Figure 8.**
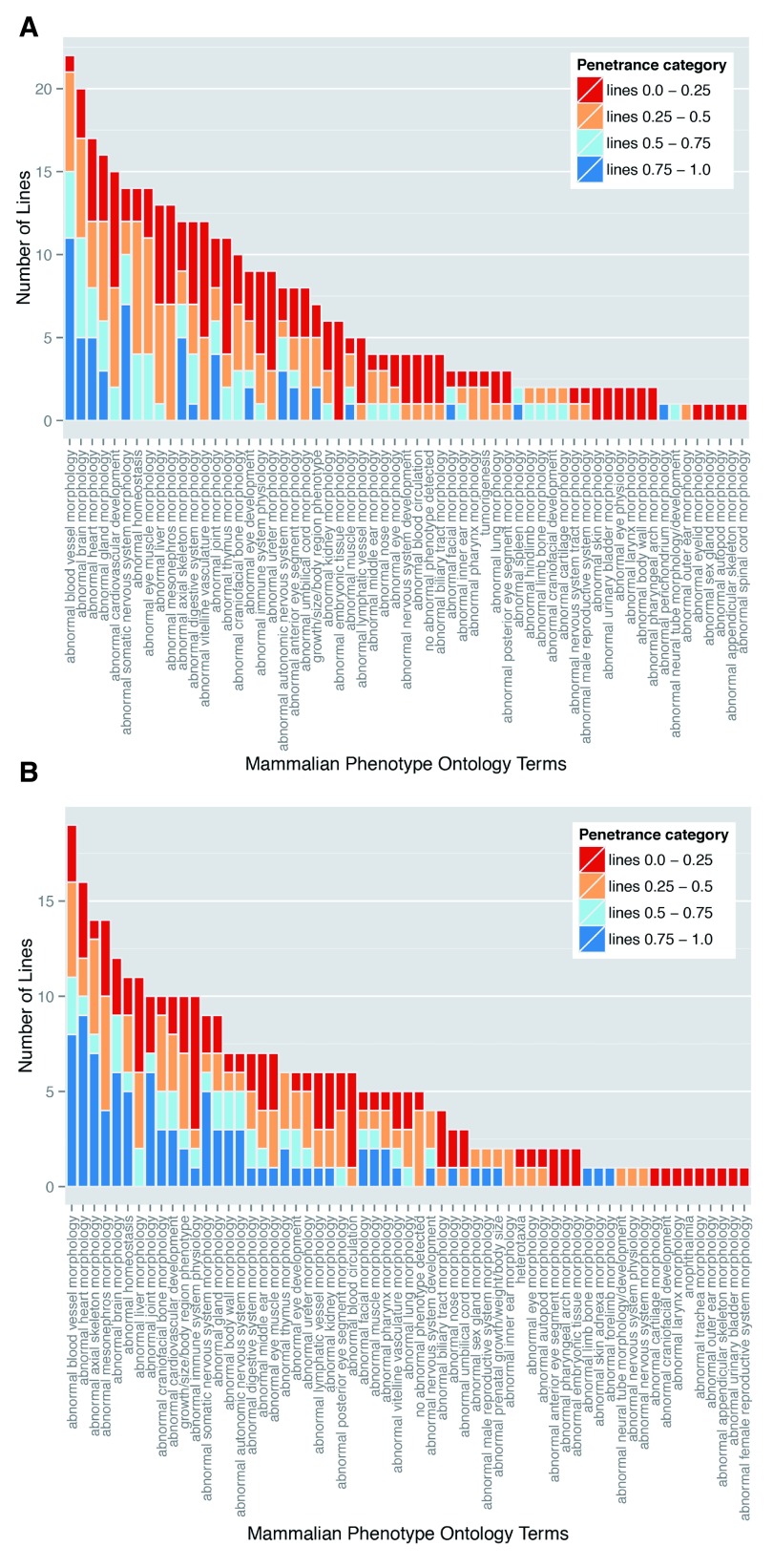
Influence of allele type on prevalence and penetrance of individual phenotypes in mutant embryos. Data from the global analysis of the frequency of phenotype terms shown in Figure 5A was subdivided by allele type to compare tm1a ( Figure 8A) and tm1b ( Figure 8B) alleles. Data is summarised using the intermediate level ontology slim and colours indicate the number of lines falling into each of the distinct penetrance categories. The data was ordered according to line frequency and subsequently by numbers seen in the penetrance categories.

Of the 42 mutant lines studied, 22 contained the tm1a insertion allele, compared with 20 containing exon deletions (19 tm1b and 1 CRISPR). With either group, blood vessel, heart and brain morphology remain amongst the most commonly observed abnormalities. There is however a clear difference in phenotype penetrance between the two groups: phenotypes are significantly less penetrant with tm1a alleles (compare Figure 5B with Figure 8A and B).

### Phenotyping embryos required new MP terms

**Figure 9.**
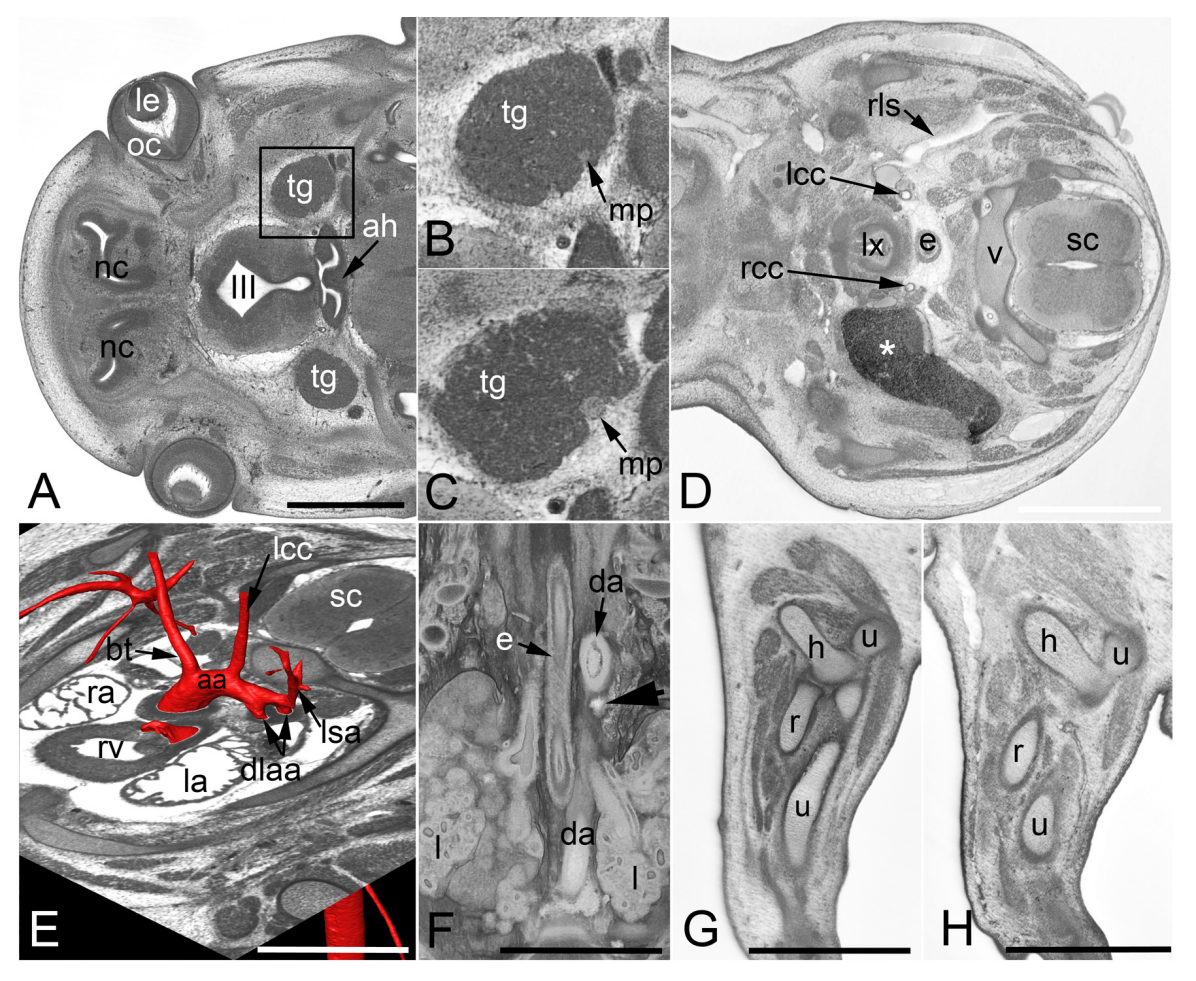
Examples of new MP phenotypes. **A**– **C**. “Thin motoric part of trigeminal nerve”. Original HREM sections through the head of a *Polb ^-/-^* embryo ( **A**, **B**) and a control ( **C**). Box in A indicates section displayed in **B**. **D** “Blood in lymph vessels”, as appearing in an original HREM section through the neck of a *1700067K01Rik ^-/-^* embryo. Note the blood filled left lymph sac (asterisk). Use the right sided lymph sac (rls) as a control. **E**. Double lumen aortic arch. Surface model of the great intrathoracic arteries on top of an original HREM section of a *Pdzk1 ^-/-^* embryo. (Compare with 17). **F**. “Intramural bleeding in blood vessel wall” (arrowhead) in the descending aorta (da) of an *Akap9 ^-/-^* embryo from the DMDD pilot study ^9^. Coronal section through a volume model. **G**– **H**. “Abnormal elbow joint morphology” Sagittal sections. Normal situation in a control ( **G**). Fusion of humerus (h) und ulna material (u) in an *Atp11a ^-/-^* embryo. aa, aortic arch; ah, adenohypophysis; bt, brachiocephalic trunk; da, descending aorta; dlaa, double lumen aortic arch; e, esophagus; h, humerus; l, lung; la, left atrium; lcc, left common carotid artery; le, lens; lsa, left subclavian artery; lx, larynx; mp, motoric part of trigeminal nerve; nc, nasal cavity; oc, optic cup; r, radius; ra, right atrium; rcc, right common carotid artery; rv, right ventricle; sc, spinal chord; tg, trigeminal ganglion; u, ulna; v, vertrebral body; III, 3rd ventricle; Scale bars: 1 mm.

Adoption of a formal, standardised ontology for scoring abnormalities provides an essential framework for analysing the data and facilitating structured search enquiries. However, during the course of the DMDD programme and its pilot study ^9^, it became clear that additional terms were required in order to adequately describe abnormalities in embryo, as opposed to adult structures. A further outcome of the DMDD study has therefore been the creation of 142 new MP terms to accommodate the range of abnormalities we have observed ( Table 7). These include, for example, thin motoric part of the trigeminal nerve (MP:0013928; http://www.ontobee.org/ontology/MP?iri=http://purl.obolibrary.org/obo/MP_0013928), blood in lymph vessels (MP:0013971; http://www.ontobee.org/ontology/MP?iri=http://purl.obolibrary.org/obo/MP_0013971), double lumen aortic arch (MP:0013981; http://www.ontobee.org/ontology/MP?iri=http://purl.obolibrary.org/obo/MP_0013981), abnormal elbow joint morphology (MP:0013945; http://www.ontobee.org/ontology/MP?iri=http://purl.obolibrary.org/obo/MP_0013945), and intramural bleeding in blood vessel wall (MP:0014020; http://www.ontobee.org/ontology/MP?iri=http://purl.obolibrary.org/obo/MP_0014020) ( Figure 9).

## Discussion

Since approximately one third of gene knockouts in the mouse prove to be embryonic or perinatal lethal ^1– 3^, further study of such lines offers a unique opportunity to better understand the genetic regulation of embryo development and identify genetic determinants of congenital abnormalities. The data accumulated during three years of the DMDD programme provide the first opportunity to study in detail the identity, range and prevalence of morphological abnormalities in such mutants and offer a window on the opportunities (and pitfalls) such systematic studies present.

The current analysis is restricted to a single developmental stage (E14.5) when most organ systems of the embryo have developed their definitive fetal appearance and the body plan is broadly similar to that of the adult mouse. Whilst this provides obvious practical advantages for a systematic, high throughput phenotyping programme, it is of course an arbitrary choice with respect to the time course of individual gene function and the consequences of gene ablation. Indeed, about 60% of the lethal lines entering the DMDD pipeline fail to provide homozygous mutant offspring by E14.5, with half of those causing lethality prior to E9.5 [see also 2]. The data here therefore comes from a subset of lethal lines. Furthermore, phenotypes observed at a single time point most likely combine more immediate consequences of individual gene loss with more distant or secondary consequences. Teasing out the role of regulative or compensatory changes from primary effects of gene loss is likely to be difficult. Despite these caveats, there are, nevertheless, several striking findings that emerge from detailed phenotype analysis.

Our finding that some manifestation of edema (generally subcutaneous) is the most common phenotype could indicate an unappreciated complexity in the genetic controls regulating fluid balance or tissue integrity of vascular or lymphatic components. Edema may also represent a common outcome for a wide range of pathophysiological perturbations, as has been proposed for the association of non-immune hydrops fetalis with human fetal loss ^11, 12^. The prevalence of cardiovascular defects is also consistent with the well established finding that cardiac abnormalities are the most common congenital defect in human newborns ^13^. Some caution is necessary in considering the mouse data, since as we have shown, a significant proportion of cardiovascular phenotypes comprise apparently minor alterations in blood vessel topology, the impact of which on normal development remains unclear. However, in addition to these, the lines we have studied show a range of severe abnormalities in cardiac structure that are both relatively prevalent and mirror the range of congenital abnormalities seen in humans. Despite the largely random selection of genes studied in screens such as DMDD, their identification as embryonic lethal therefore provides a dramatic enrichment for potential cardiac developmental disease alleles.

Phenotypes affecting neural tissue also prove to be relatively prevalent in mutant embryos. We are limited in the present analysis to identifying a subset of neural deficits readily identified from HREM imaging. This restricts identifiable phenotypes to relatively gross alterations in brain and neural tube morphology, or changes affecting major nerves. Amongst the latter, the frequency with which abnormalities affecting the hypoglossal nerve have been detected is perhaps not so surprising, since these (like abnormalities detected in the motoric portion of the trigeminal nerve) may compromise suckling and lead to perinatal lethality.

The multiplicity of phenotypes frequently detected in individual mutant embryos is not unexpected, given the nature of a single time point screening procedure, combined with the likely pleiotropic effects of individual gene loss. However, the most striking and surprising finding to emerge from the DMDD phenotype data is that virtually all phenotypes are incompletely (and frequently poorly) penetrant, despite the use of the isogenic C57BL/6N mouse strain. Combined with the observation of overlapping but distinct spectra of phenotypes between individual embryos from a single line, these findings are challenging to understand, and at a minimum point towards unknown stochastic components affecting the etiology of each phenotype or the compensatory responses they elicit ^2^. They also demonstrate that efforts to identify linkage between mouse embryo phenotypes and human developmental disease are likely to require sophisticated bioinformatic analysis beyond the obvious issues raised by species differences in anatomy and physiology.

The observation of a small number of phenotypes amongst the wild type litter mates of the homozygous mutants raises the important question: why are phenotypes detected in genetically wild type embryos? We think there are several possible explanations. One possibility is that the C57BL/6N mouse strain used for engineering knockout lines carries a “background load” of abnormalities, previously unappreciated. Ours is the first systematic study on sufficiently large scale and employing sufficiently high-resolution imaging to detect such abnormalities. None of the phenotypes we have identified show a high penetrance across both mutants and wild types of a mutant line and do not therefore suggest themselves as strain-specific abnormalities. Another possible explanation is that abnormalities arise as a consequence of *de novo* mutation. Lastly, at least with the less profound abnormalities, it is possible that some phenotypes may prove to be outliers on spectrum of normal morphological variation and should not be considered genuine abnormalities. This highlights an important issue confronting phenotyping studies: the dearth of large-scale and systematic studies examining normal embryo morphology that can set a reliable benchmark for distinguishing abnormalities from normal variation. In this light, phenotype data may need revision as cumulative experience with the C57BL/6N and other mouse strains improves our ability to distinguish abnormalities from normal variation amongst wild types.

Our study has identified a small number of apparent abnormalities common to both homozygous mutant embryos and wild-type controls from the C57BL/6N mouse strain and which have therefore been excluded from the phenotyping procedure. These include splitting of the tail tip, persistence of the craniopharyngeal duct with associated fenestration of head bones and the presence of vesicles in the lens of the eye ( Figure 4, panels C–E). Apart from these, our data offers no clear evidence for other “background” phenotypes associated with either the C57BL/6N genetic background or with individual mutant lines. Overall, we consider that neither the frequency, prevalence nor nature of the phenotypes identified in wild type embryos impact significantly on the assignation of phenotypes amongst the homozygous mutant embryos.

Two other factors in our study might affect interpretation of the mutant phenotype data. 11 of the 42 lines examined in our study were judged subviable at weaning, rather than lethal. This number is too small to support meaningful comparison of the phenotypic spectrum between subviables and lethals. It is tempting to speculate that a difference in phenotype penetrance might underlie the difference in viability between the two groups, but there is no evidence to support this from the DMDD study so far (see Supplementary Figure 4). Even if a difference in penetrance was detected between lethal and subviable lines, interpreting its significance is far from simple as it raises an important and unresolved question: which phenotypes are responsible for embryo death? Many profound abnormalities that we detect may be compatible with life; equally, lethality may result from subtle structural changes. Without knowing which of the scored phenotypes are likely to cause lethality, it will be difficult, if not impossible, to establish if differences in their penetrance distinguish subviable from lethal lines. Add to this the additional difficulty that dams have a propensity to eat newborns that are not thriving well and there is a further complication in interpreting the data.

The lines we have studied fall roughly equally between those containing an insertion into the targeted gene (tm1a alleles) and those in which recombination has removed both a gene exon and the neomycin selection cassette (tm1b alleles). Interestingly, our data clearly reveals that tm1b alleles show greater penetrance of phenotypes than those containing the tm1a insertion. This may reflect the potential of tm1a alleles to be hypomorphic, and might also be influenced by their retention of the neo selection cassette.

It is also worth noting the several practical lessons which have become evident through the course of DMDD studies and which may be of value for similar embryo phenotyping programmes. The most pressing of these is basing phenotype detection on comparison of each mutant embryo with an appropriately staged normal counterpart ^14^. Embryos harvested at E14.5 vary markedly in their developmental progress and many tissues and organs are actively remodelled during this period. This is most obvious for the topology of the intestine, the position of the palatal shelves and the interventricular communication between left and right sides of the heart. Only with precise developmental staging is accurate phenotyping of these features possible ^8^.

Whilst the precise range and detail of phenotypes that can be scored will necessarily be dictated by the nature of the imaging modality and the method of phenotype identification (compare, for example 15, 16, with the manual annotation used in the present study), a common challenge is the development of protocols to minimise occurrence or subsequent scoring of apparent abnormalities that are more likely artefacts of sample preparation or processing. These can range from the more obvious ruptures of the embryo skin or damaged external features during dissection, to tissue shrinkage or swelling (causing organ deformation) as a result of dehydration, fixation or embedding. Finally, the power of phenotypic screens such as DMDD to inform our understanding of developmental disease rests heavily on the detail with which abnormalities are scored. However, the very complexity we have seen this generates makes it all the more urgent to distinguish phenotypes not just through the nature of the morphological abnormality, but through its capacity, individually or in concert with others, to compromise subsequent fetal survival.

## Data availability

Dataset 1 Zenodo: 10.5281/zenodo.163506 ^18^

Dataset 2 Zenodo: 10.5281/zenodo.268899 ^19^

The cumulative list of all scored phenotypes analysed in this study is presented in Dataset 1 (homozygous mutants) and Dataset 2 (wild type embryos). The intermediate and high level slims of the MP ontology used in the analysis are presented in Supplementary table 2 and Supplementary Table 3. All data used in this study is also available from the DMDD web site ( https://dmdd.org.uk) where phenotype annotations are available in tabular format by embryo and by line. In addition, they are identified at their appropriate locations within each 3D dataset of embryo images, which can be viewed in all three orthogonal section planes.

## Supplementary material

**Supplementary Figure 1: Embryo Homozygous mutant embryo numbers analysed for each mutant line.**

The number of annotated embryos scored for each of the 42 lines was used to plot the variation in numbers of embryos analysed per line.

**Supplementary Figure 2: Distribution of homozygous mutant embryo phenotypes amongst DMDD mutant lines (high level MP ontology slim).**

Data from the global analysis of the frequency of phenotype terms (see Materials and Methods) is plotted to show the penetrance of phenotypes scored for each line, indicated by a colour gradient from light yellow (no penetrance) to dark red (100% penetrance). Each line is labelled after the symbol of the gene disrupted (see also Table 1), and the number of homozygous mutant embryos analysed for each line is shown. Phenotype annotations are summarised using the high level DMDD ontology slim.

**Supplementary Figure 3: Distribution of homozygous mutant embryo phenotypes amongst DMDD mutant lines (intermediate level MP ontology slim).**

As in Supplementary Figure 2, except that phenotype annotations are summarised using the intermediate level DMDD ontology slim.

*Supplementary Figure 4: Penetrance of mutant embryo phenotypes in lethal and subviable mutant lines*.

Data from the global analysis of the frequency of phenotype terms (see Materials and Methods) was plotted using embryos of the same genetic background ( *C57BL/6N;C57BL/6NTac*) to show the number of lines falling into each of the observed phenotype categories (using the MP ontology intermediate slim). Each of the distinct penetrance categories is colour coded and data is ordered according to line frequency. (A) shows the penetrance distribution in the combination of lethal and subviable lines; (B) shows the equivalent plot for lethal lines alone.

**Supplementary Table 1A and 1B: Embryo phenotypes, organised by frequency.**

The data from the global analysis of the frequency of phenotype terms (see Materials and methods) is presented in a structured fashion showing the relationship between Mammalian Phenotype Ontology terms included in the DMDD high level ontology slim, the DMDD intermediate level ontology slim, and the original annotation terms. The first three columns list the ID, term and frequency of DMDD high level ontology slim terms, columns 4–6 list the ID, term and frequency of the intermediate level ontology slim terms that cluster under the high level term listed in column 1, and for each intermediate level term the annotation phenotype terms are shown in order of frequency in columns 7–9. Column 10 lists the lines in which the phenotype listed in columns 7 was observed. The table rows are ordered according to the frequency of the high level ontology terms, intermediate ontology terms and original annotation terms. Table 1A: homozygous mutant embryos; Table 1B: wildtype embryos.

Table 1A:

Table 1B:

*Supplementary Table 2: High level MP ontology slim used by DMDD.*

A list of the Mammalian Phenotype Ontology IDs and names of terms selected as the high level ontology slim.

*Supplementary Table 3: Intermediate level MP ontology slim used by DMDD.*

A list of the Mammalian Phenotype Ontology IDs and names of terms selected as the intermediate level ontology slim.

*Supplementary Tables 4 and 5: All embryo phenotypes from lethal and sub-viable lines scored by DMDD to date.*

The tables list the annotation data that is the basis of the study. For every annotation the gene symbol, MGI_ID, allele symbol, DMDD_ID, MP term, ID and name is listed. In some cases the same MP term is listed more than once for a specific embryo (DMDD_ID), indicating the phenotypic abnormality was observed more than once in that embryo.

Supplementary Table 4: homozygous mutant embryos

Supplementary Table 5: wild type embryos
