# Supplementary figures and images for: Highly variable penetrance of abnormal phenotypes in embryonic lethal knockout mice
Source: Wellcome Open Res. 2017 Feb 27;1:1. Originally published 2016 Nov 15. [Version 2] doi: 10.12688/wellcomeopenres.9899.2 (PMC5159622; doi:10.12688/wellcomeopenres.9899.2)

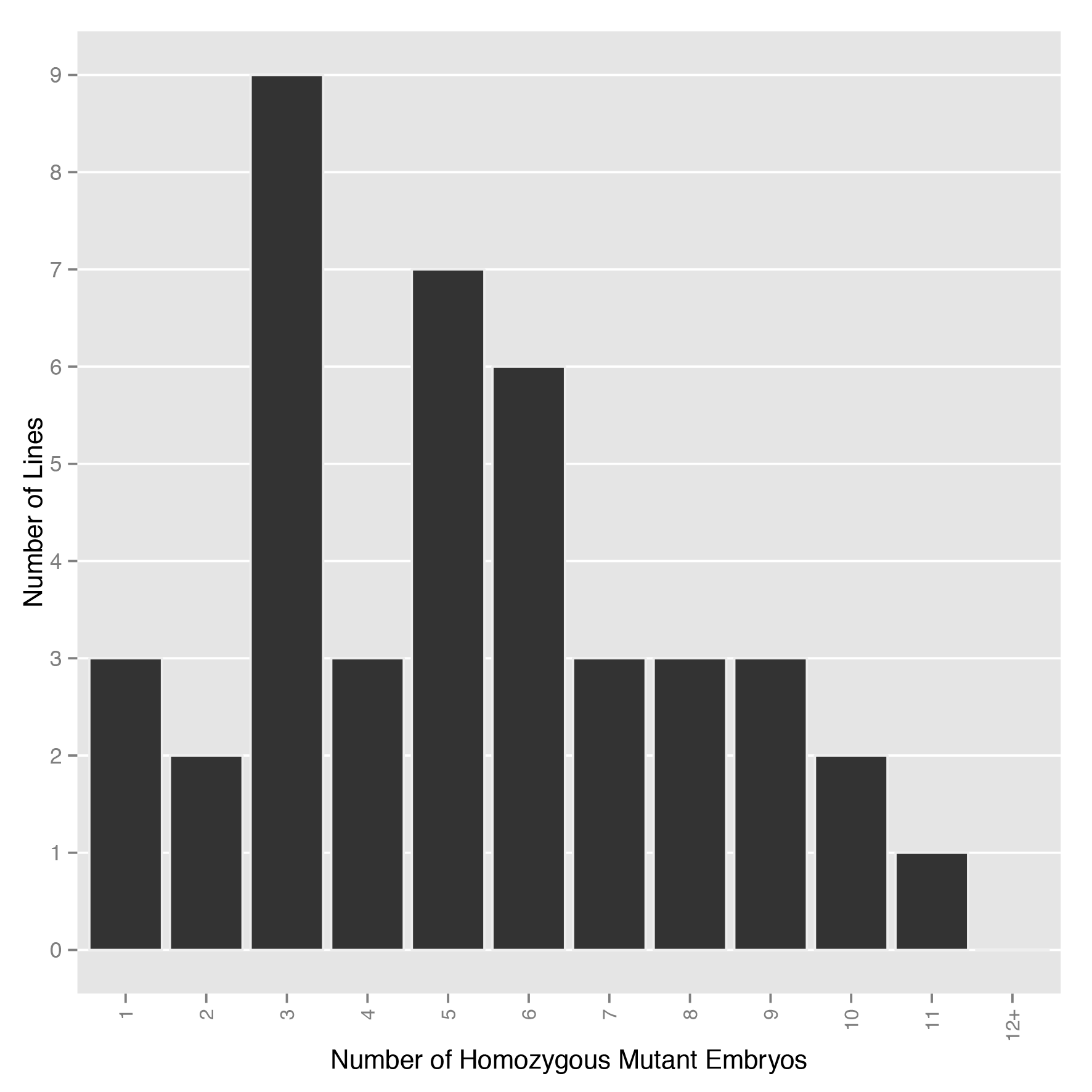

Supplement: Supplementary file 1 [file wellcomeopenres-1-11724-s0000.tgz › 3e9b93b8-6003-4d52-90cb-47138ca4d78d.tif]

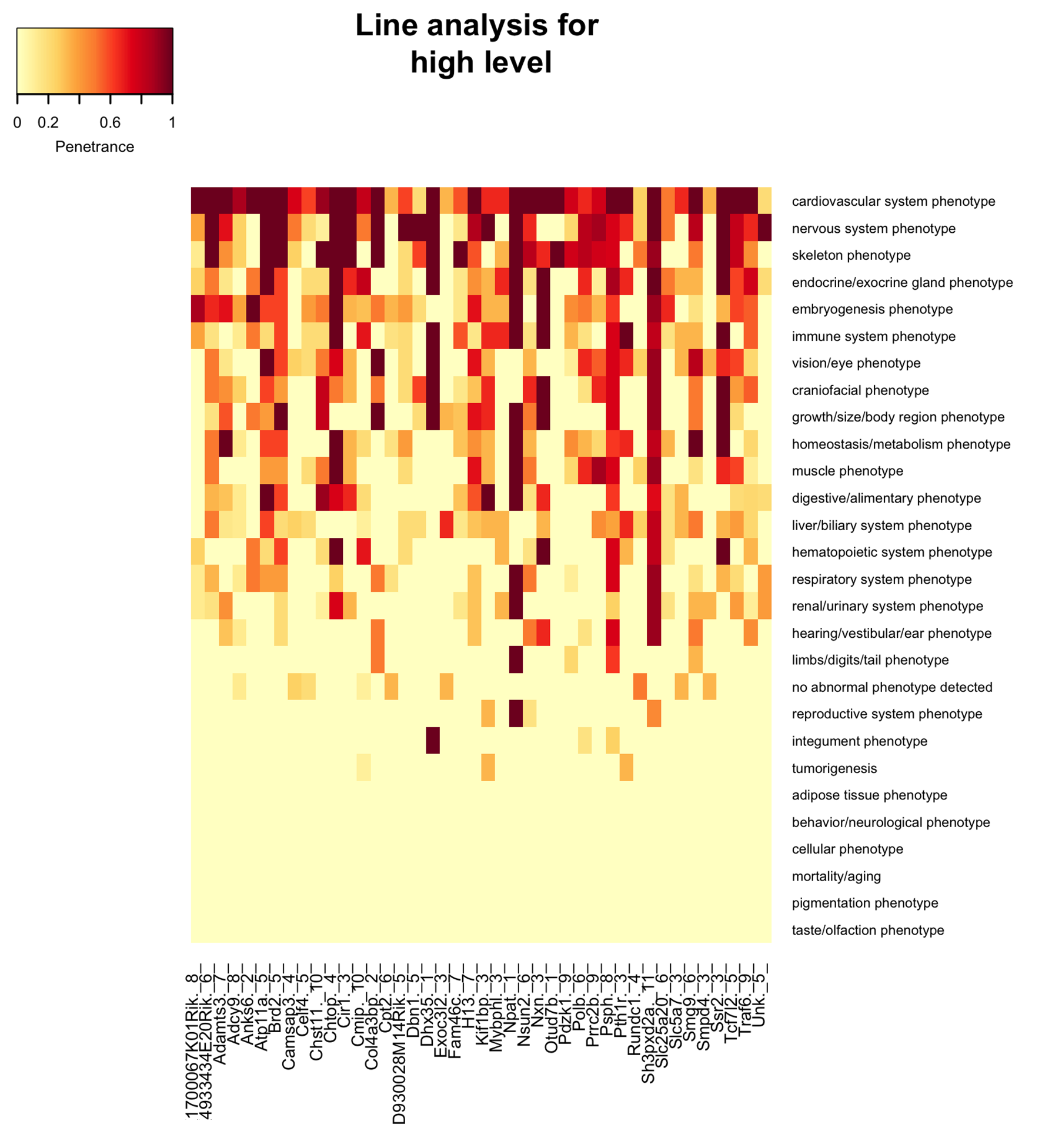

Supplement: Supplementary file 2 [file wellcomeopenres-1-11724-s0001.tgz › e4b43a57-c0e1-40b6-89e4-9f3c8c5fb780.tif]

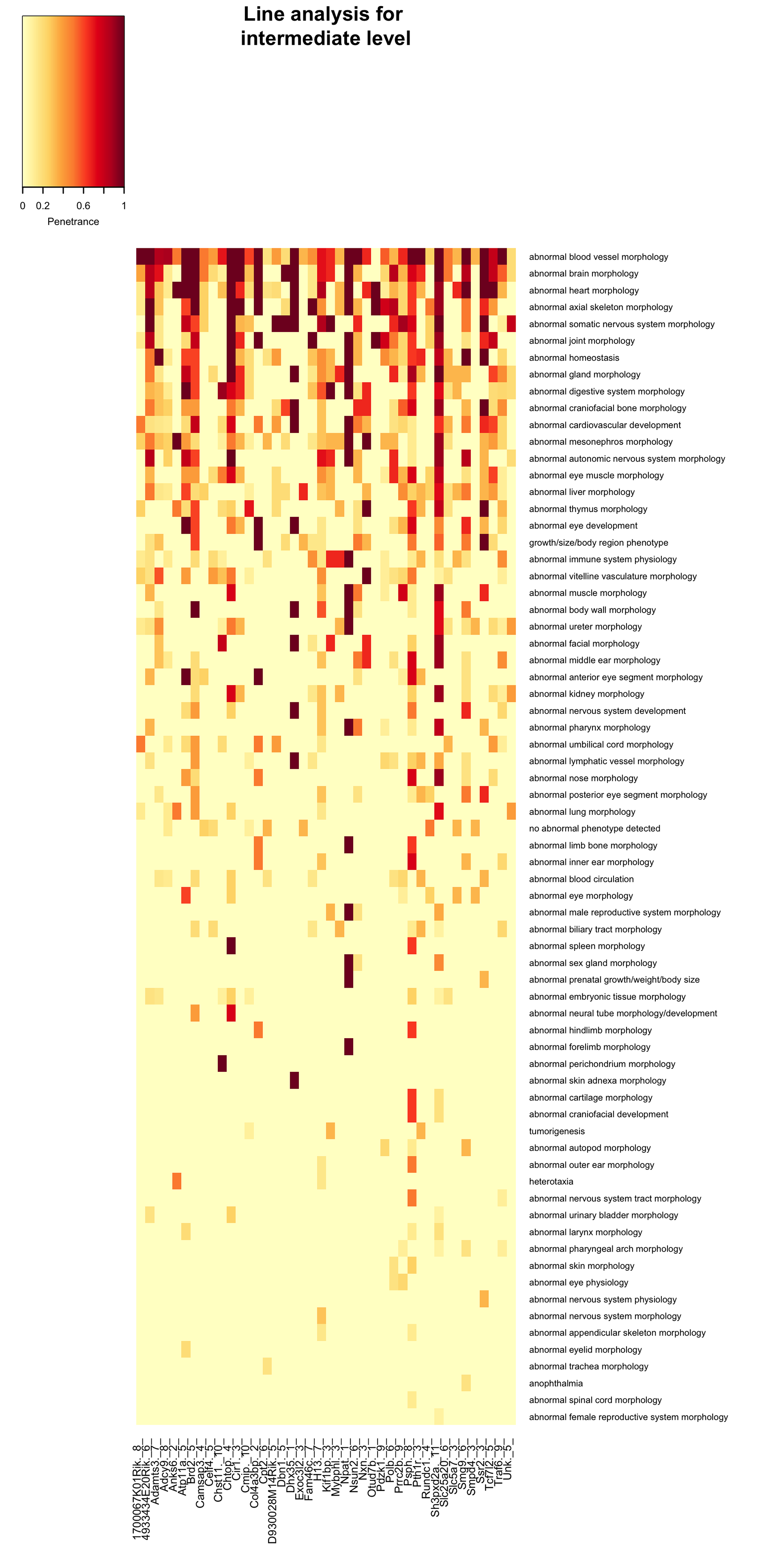

Supplement: Supplementary file 3 [file wellcomeopenres-1-11724-s0002.tgz › 37bdc0f8-b48b-42df-a0b2-b8fd464984a5.tif]

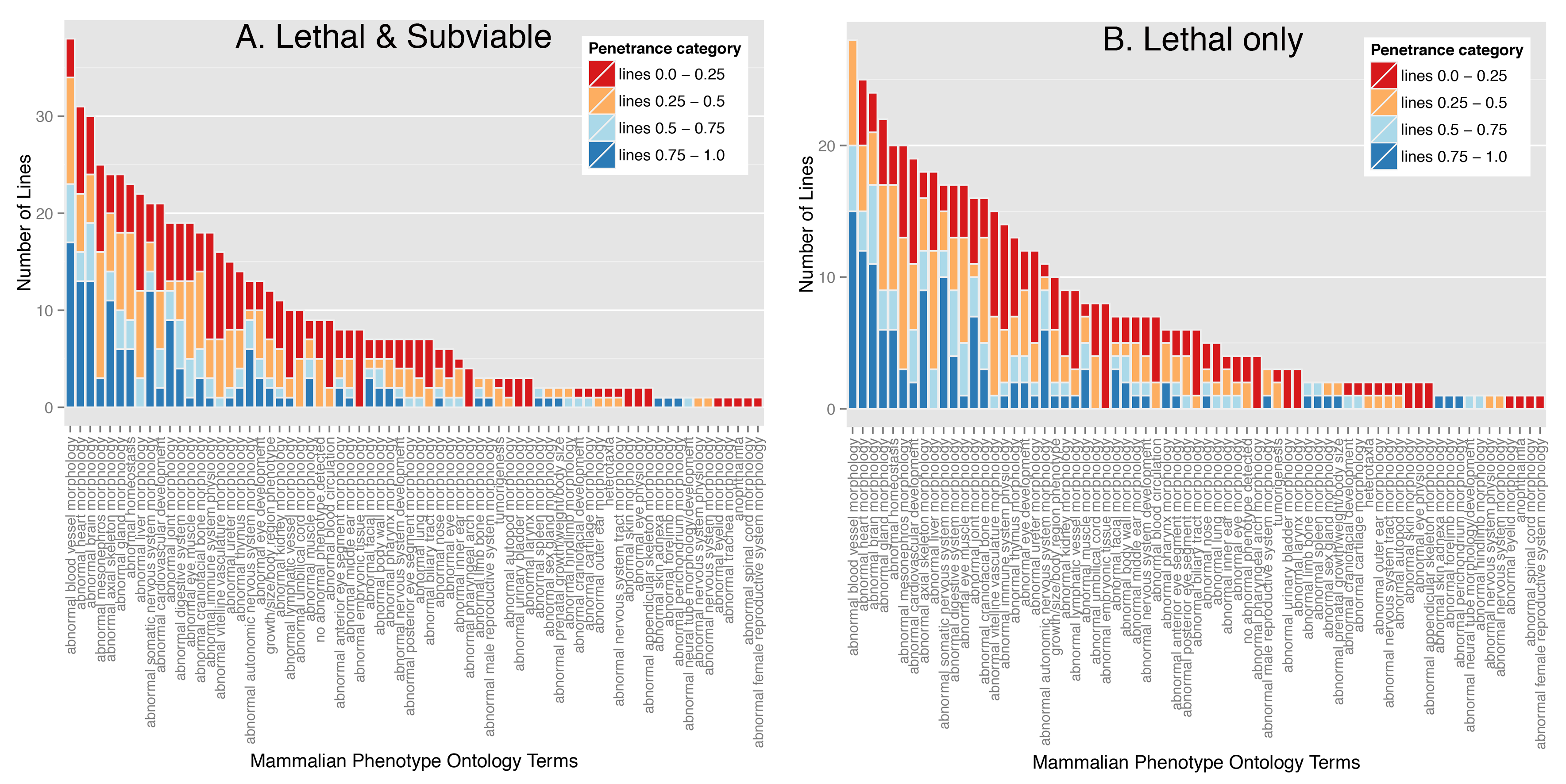

Supplement: Supplementary file 4 [file wellcomeopenres-1-11724-s0003.tgz › 55104fd8-c325-4a36-9058-96dc379d0e37.tif]
