# Supplementary material for: Highly variable penetrance of abnormal phenotypes in embryonic lethal knockout mice
Source: Wellcome Open Res. 2017 Feb 27;1:1. Originally published 2016 Nov 15. [Version 2] doi: 10.12688/wellcomeopenres.9899.2 (PMC5159622; doi:10.12688/wellcomeopenres.9899.2)
Supplement: Peer review history

# Peer review: Referee response for version 1

**DOI:** 10.21956/wellcomeopenres.10670.r18405

**Published:** 2016-12-12

**Authors:** Lydia Teboul

**Affiliations:** 1 The Mary Lyon Centre, Medical Research Council Harwell Institute, Didcot, UK

The Deciphering Mechanisms of Developmental Disorders consortium presents a systematic study of the morphology of mutant embryos from 42 lines developed in the frame of the International Phenotyping Consortium. The lines chosen for this study were selected as they are homozygous lethal or subviable at weaning but viable at E14.5.

 The authors employ High Resolution Episcopic Microscopy to capture 3D images of the embryos, providing exquisitely detailed documentation of embryo morphologies. They exploit this rich dataset with a systematic and in depth annotation of morphological defects which they record using appropriate levels of MP terms.

 The result is a survey of impressive scope in terms of annotation depth and volume of data, and a superb effort of data organisation and analysis so the great complexity of the dataset can be distilled to overall observations and discussion points. This organisation effort yielded a really useful framework for systematic analysis of the morphology of mouse mutant of that stage.

 The authors conclude that a salient point of the work is the great variability of penetrance of the morphological phenotypes they find among these mutant embryos of the same isogenic genetic background.

 Although the variable expressivity of phenotype between different individuals of a same mutant line isn’t a new concept, the unexpected result of the study is the extend to which phenotypes (even when grouped in broad categories such as “organ affected”) vary in penetrance, albeit that these mutants share the broadest of phenotype which is lethality.

 However, the authors restrict their analysis to the variability amongst mutants and they mention in the discussion an on-going systematic analysis of WT embryos, which will provide key information to put in context the observations collated in this article.

 Whereas the article is an excellent effort of presenting a complex dataset with clarity and granularity and documenting variability of morphology amongst samples, the data presented do not allow the reader to identify the reason(s) of this variability in the absence of key information. Three major points should be addressed:

- The authors made the unusual choice of not presenting baseline data on the morphology of wild-type mutants (littermates) produced in the study. Such data, surveying significant groups of control embryos, would be essential to establish the link between mutations and described phenotypes. In the absence of this data, any reference to a causal link between phenotypes and mutation should be removed from the article.
- Both targeted traps (tm1a) and null (tm1b and CRISPR induced deletions) alleles are employed in the study. Both the presence of a selection cassette and the unpredictability of efficiency of trapping cassette(s) could form the basis of at least some of the variability shown in this study. An evaluation of variability (particularly using slim terms) within each of these 2 groups of alleles would help to address this point.
- Subviable lines show by definition a partially penetrant phenotype and contribute to a quarter of the mutant studied. An evaluation of variability (particularly using slim terms) within lethal and subviable as separate alleles groups would discriminate whether variability of morphology is particularly occurring among subviable lines.

 

 Minor points:

- Methods should detail information that permit the appraisal of materials used in the study, detailing the genetic background of stem cells and animals employed for germline transmission, and further breeding, including whether homozygotes were used to produce embryos to analyse subviable lines.
- Methods should outline the steps taken to limit manual annotation variability (i.e. secondary calling or benchmarking between annotators).
- All titles and text should precisely detail when lethal or both lethal and subviable mutations are presented.

I have read this submission. I believe that I have an appropriate level of expertise to confirm that it is of an acceptable scientific standard, however I have significant reservations, as outlined above.

# Response

**Published:** 2017-03-06

**Authors:** Tim Mohun

**Affiliations:** The Francis Crick Institute, UK

**The authors made the unusual choice of not presenting baseline data on the morphology of wild-type mutants (littermates) produced in the study. Such data, surveying significant groups of control embryos, would be essential to establish the link between mutations and described phenotypes. In the absence of this data, any reference to a causal link between phenotypes and mutation should be removed from the article.**

 We have included the wild type phenotype data in the revised version of the manuscript (see the detailed response to Rosenthal/Murray for more details).

**Both targeted traps (tm1a) and null (tm1b and CRISPR induced deletions) alleles are employed in the study. Both the presence of a selection cassette and the unpredictability of efficiency of trapping cassette(s) could form the basis of at least some of the variability shown in this study. An evaluation of variability (particularly using slim terms) within each of these 2 groups of alleles would help to address this point.**

 The revised manuscript now includes separate analysis of phenotypes for the 22 tm1a alleles compared with 20 complete nulls (19 tm1b and 1 CRISPR). With either allele, blood vessel, heart and brain morphology remain amongst the most commonly observed abnormalities. However, with such relatively small numbers, we feel there is little more that can usefully be concluded from comparison of individual phenotype prevalence, since this will be heavily influenced by the distinct gene identities within each allele group. In contrast, there is a clear difference in phenotype penetrance between the two groups: phenotypes are clearly more penetrant from tm1b alleles (see new Figure 8A and 8B). We presume that this reflects the fact that whilst mutations based on tm1a alleles have the potential to be hypomorphic, those converted from tm1a to tm1b contain an exon deletion (and no longer carry the neo selection cassette).

**Subviable lines show by definition a partially penetrant phenotype and contribute to a quarter of the mutant studied. An evaluation of variability (particularly using slim terms) within lethal and subviable as separate alleles groups would discriminate whether variability of morphology is particularly occurring among subviable lines.**

 We presume that the reviewer is wondering whether the difference between lethal and subviable lines is a result of differing degrees of penetrance of phenotypes that result in embryo death. Answering this point is not as simple as it might appear as it touches on a much more profound issue raised by studies such as ours. Whilst we are able to distinguish a remarkable number of different structural abnormalities by virtue of the resolution HREM imaging affords, it may not be at all clear which of these results in embryo lethality. Many profound abnormalities may be compatible with life and lethality may also result from structurally subtle changes. Without knowing which of the scored phenotypes are likely to cause lethality, it will be difficult if not impossible to establish of differences in their penetrance distinguish subviable from lethal lines. Add to this the additional difficulty that dams have a propensity to eat newborns that are not thriving well and there is a further complication in interpreting the data.

 We have nevertheless reexamined the phenotype data in order to compare the results separately for lethal and subviable lines (new Supplementary Figure 4). From this it is clear that there is insufficient data from subviable lines to draw unequivocal conclusions. Overall, the approximate prevalence of particular phenotype terms (using the intermediate slim) appears broadly similar to that of lethals, but for most of these, the numbers of affected lines are too few to make useful estimates of penetrance.

**Minor points:**

 1. Full details of genetic background and mutant allele are now provided for each line (revised Table 1).

 2. All phenotyping was performed according to a standardised and sequential procedure, as mentioned in Material and methods. The data from each embryo was independently reviewed by a second anatomist and any discrepancies resolved by joint agreement.

 3. We have amended titles and text to ensure that the distinction between lethal and subviable lines is clear where necessary.

# Peer review: Referee response for version 1

**DOI:** 10.21956/wellcomeopenres.10670.r18334

**Published:** 2016-12-08

**Authors:** Nadia Rosenthal, Steve Murray

**Affiliations:** 1 National Heart and Lung Institute, Imperial College London, London, UK; 2 The Jackson Laboratory, Bar Harbor, ME, USA

This manuscript describes the findings of the DMDD consortium, analyzing 42 lethal and subviable genes at E14.5 using high-resolution 3D imaging (HREM) coupled with detailed annotation of the specific phenotypes revealed. The level of granularity in the scoring of the phenotypes is a major strength of the paper, and reflects the deep and unique expertise of the team. This has facilitated the discovery of widespread variable penetrance in mutant embryos at a level of detail not previously described. Furthermore, the effort to organize the MP into a series of “slims” is quite useful for organizing the calls into easier to analyze groups, and such work will likely benefit other groups such as the IMPC. 

 The manuscript is clearly written and, importantly, goes to great lengths to ensure full access to all data. In addition to minor issues detailed below, there are two major gaps, however, that must be addressed.

1. There is no description of the number of control embryos screened or the incidental rate of hits for each phenotype in the DMDD list. Given the focus of the paper on the variability of phenotype penetrance and the number of phenotypes with an “n=1”, it is impossible to draw conclusions without this information. While the authors allude to a manuscript in preparation, it is actually essential data for this paper.
2. Similarly, there is no description of how the authors account for global developmental delay in mutants, which can lead to many “phenotypes” that are merely the result of slowed/retarded development or variability in developmental timing between litters. For example, at E14.5, one would expect a high rate of cleft palate in mutants that have some level of overall delay, or in entire delayed litters, as the palate is elevating and fusing at that time point. This raises the following questions: are controls from each litter collected? How is uniform staging assured? Are “delayed” embryos compared to a stage-matched control? Again, the authors allude to another manuscript, but some of this information needs to be included here to assure the MP calls do not have trivial explanations.

 

 Minor points:

1.  While the brief description of the animal resource and use of website citation is acceptable, given the main finding of variable penetrance, the authors should make a point of describing the isogenic genetic background and the nature of the alleles (tm1a or tm1b) in the methods and results.
2. It’s not entirely clear if this was a set of 42 genes that were lethal/subviable at wean, or if this was a select set of lethal genes that were viable/subviable (present) at E14.5. Given the comments in the discussion about lines lethal at E9.5 or earlier, I assume the latter. This should be spelled out.
3. Mouse gene symbols should be italicized.
4. Apart from Table 1, the tables are too large and make reading a PDF a somewhat painful process. These might not be easily compressed, so most of the information should be moved to a supplemental file.

We have read this submission. We believe that we have an appropriate level of expertise to confirm that it is of an acceptable scientific standard, however we have significant reservations, as outlined above.

# Response

**Published:** 2017-03-06

**Authors:** Tim Mohun

**Affiliations:** The Francis Crick Institute, UK

**There is no description of the number of control embryos screened or the incidental rate of hits for each phenotype in the DMDD list. Given the focus of the paper on the variability of phenotype penetrance and the number of phenotypes with an “n=1”, it is impossible to draw conclusions without this information. While the authors allude to a manuscript in preparation, it is actually essential data for this paper.**

 The revised manuscript now includes the complete phenotype data obtained for 114 wild type embryos. This comprises 56 phenotype calls, affecting 32 embryos, originating from 28 lines (revised Tables 1, 2B and Supplementary Table 5). 21 of the 56 phenotype calls (38%) are accounted for by only 6 embryos, (indicating the skewing effect of a small number of abnormal embryos), most affected embryos showing only a single phenotype. This is in marked contrast to the finding of many different phenotypes in individual mutant embryos. The phenotypes of wild types vary in character, ranging from apparently minor differences (e.g. in blood vessel morphology) to a few major abnormalities (e.g. absent kidney). Each one is rare amongst the population of wild type embryos analysed and affects only a single wild type embryo within the line. Only 10 phenotypes (15 phenotype calls) overlap between mutant embryos and their wild type siblings and these affect only 10 of the 41 lines for which wild type embryos have been assessed (Table 3). As we discuss in the revised Results and Discussion sections, these data raise 3 related questions: Why are phenotypes detected in genetically wild type embryos? Are there “background” phenotypes associated with the C57BL/6N line that contribute to the mutant phenotypes scored? Is there any evidence for “background” phenotypes associated with an individual knockout line?

 We think there are several possible explanations for finding phenotypes amongst wild type embryos. One possibility is that the mouse strain that has been used for engineering knockout lines carries a “background load” of abnormalities, previously unappreciated. Ours is the first systematic study on sufficiently large scale and employing sufficiently high resolution imaging to detect such abnormalities. Amongst the phenotypes identified, none shows significant prevalence that might be expected if it was a strain-specific abnormality. Another possible explanation is that abnormalities arise as a consequence of de novo mutation and the frequency we detect reflects the high sensitivity that results from HREM imaging. Lastly, at least with the less apparently severe abnormalities, it is possible that some of these in fact represent outliers on spectrum of normal morphological variation and should not be considered genuine abnormalities. This highlights an important issue confronting phenotyping studies: the dearth of large-scale and systematic studies examining normal embryo morphology that can set a reliable benchmark for distinguishing abnormalities from normal variation. In this light, phenotype data may need revision as cumulative experience improves our ability to distinguish abnormalities from variation amongst wild types.

 Whatever the explanation, it is clear that neither the frequency, prevalence nor nature of the phenotypes identified in wild type embryos impact significantly on the assignation of phenotypes amongst the homozygous mutant embryos.

**Similarly, there is no description of how the authors account for global developmental delay in mutants, which can lead to many “phenotypes” that are merely the result of slowed/retarded development or variability in developmental timing between litters. For example, at E14.5, one would expect a high rate of cleft palate in mutants that have some level of overall delay, or in entire delayed litters, as the palate is elevating and fusing at that time point. This raises the following questions: are controls from each litter collected? How is uniform staging assured? Are “delayed” embryos compared to a stage-matched control? Again, the authors allude to another manuscript, but some of this information needs to be included here to assure the MP calls do not have trivial explanations.**

 We believe it is important to distinguish between the effect of precise developmental stage of phenotyping and the issue of developmental retardation or delay. We can now reference the published study we mentioned that addresses these very questions (Geyer et al. 2017, J. Anat. in press). We do indeed collect wild type controls from each litter but our experience has demonstrated that precise stage matching of mutants with controls is essential to underpin accurate phenotyping. To facilitate this, we have analysed a large number of wild type embryos from the same genetic background as the that used for engineering of mutant lines. We have developed a system that can reliably distinguish five sub-stages within the span of Theiler stages 21 to 22 that are collected during E14.5, enabling us to compare each mutant embryo against precise, developmental stage-matched controls. Careful study and comparison of these has identified those changes (such as fusion of palatal shelves) which occur during the window of development that we observe. By combining qualitative comparisons with quantitative morphometry and statistical analysis, we are able to distinguish what can be considered genuine abnormalities from features that show either rapid developmental change or significant variability in the developmental timing of their appearance.

 A more precise staging system also allows us to phenotype homozygous mutant embryos accurately, even though they frequently show some developmental delay, since we are able to compare them to controls at the equivalent stage of development. It also allows us to score instances of heterochrony where this affects individual (or a limited subset of) organs or tissues. By analysing a large number of wild type embryos harvested at E14.5, we have identified the spread and distribution of individual developmental sub stages that might be expected, and on this basis have a robust, statistical definition for global developmental retardation. Our studies do not allow us to identify why such retardation is relatively common amongst mutant embryos, but do offer some interesting pointers that we have commented upon. Retardation is, for example, much more common in mutant embryos showing cardiovascular defects (Geyer et al. 2017, J. Anat. in press). Furthermore, a surprisingly large proportion of mutants show abnormalities in their placental structure, and this may perhaps impact on their overall growth and development (unpublished data).

**Minor points**

 1. The genetic background and details of each allele are now included in revised Table 1. 39 of the 42 lines analysed are on an identical background (C57BL/6N;C57BL/6NTac). 22 lines contain the tm1a allele, 19 contain tm1b and 1 line was produced using CRISPR.

 2. The “Embryos” section of Materials and Methods details how the 42 lines were designated as lethal or subviable at wean (P14).

 3. Mouse gene symbols have been italicised.

 4. It was not possible for the larger tables to be moved to supplementary files; this is an unfortunate limitation of the online presentation method. Wellcome Open Research requested that the tables were included as figures rather than supplementary data and we agree that it is helpful for the reader to see the nature of the data. We had hoped that the individual files could also be downloaded in their spreadsheet format to allow full interrogation but the interface does not currently allow this. We have requested this change but in the meantime large tables are now also included in supplemental spreadsheet files to allow the reader to search and filter the data as required.

# Peer review: Referee response for version 1

**DOI:** 10.21956/wellcomeopenres.10670.r17602

**Published:** 2016-12-05

**Authors:** David Brook

**Affiliations:** 1 School of Life Sciences, University of Nottingham, Nottingham, UK

We live in interesting times. Election outcomes are unpredictable. People are unpredictable and now as Wilson *et al.* report even the consequences of specific mutations are significantly less predictable than we might expect.

 The Deciphering the Mechanisms of Developmental Disorders (DMDD) programme aims to analyse 240 embryonic lethal mouse knockout lines over a five-year period to study genes essential for mouse embryonic development and survival. This paper provides the first report on results gathered thus far. Wilson et al performed a detailed assessment of morphological abnormalities at stage E14.5 in 220 embryos from 42 novel mouse gene knockout lines. High Resolution Episcopic Microscopy was used to detect abnormalities at a scale from whole organs and tissues down to individual nerves and blood vessels. They report multiple abnormalities in virtually all of the embryos studied. They generated a wealth of information; in excess of 1.6 million images including more than 700,000 transverse sections to detail the incidence of structural abnormalities in 209 of the 220 embryos analysed. Eleven of the embryos from nine different lines were apparently normal.

 To provide systematic phenotypic data Mammalian Phenotype (MP) ontology terms were used to classify abnormalities with high and intermediate levels. This allowed the authors to calculate a penetrance score for the terms in each of the mutant lines and to assign these to a quartile percentage group. Only 3 phenotypes were 100% penetrant and over half of the abnormalities had a penetrance score under 25%.    

 Approximately one third of mouse gene knockouts are lethal and 60% of lethal lines entering the DMDD programme fail to provide homozygous mutant offspring by E14.5 with half of those being lethal prior to E9.5. Thus, as the authors point out, the data presented are from a subset of lethal lines. However, the most striking aspect of this study is the variability in penetrance of virtually all of the phenotypes analysed.

 Recent studies sequencing human exome DNA has identified a high frequency of loss of function mutations. A study by Lek et al 2016 ^1^ examined more than 60,000 human exomes and reported predicted homozygous loss of function genotypes in 1775 genes. On average there are 35 homozygous gene deletions in each human. Thus the comment by Wilson et al in the present paper is particularly pertinent; relating these findings to human developmental disease will require further sophisticated analysis. It would appear that homozygous loss of function mutations are more common than previously realised and, furthermore, the consequences of loss of function mutations are much more variable than previously realised. It will not be trivial to unmask the causes of this variability. We are only just beginning to scratch the surface of understanding the consequences of loss of function mutations in both mice and humans.

 I have only one minor suggestion. On p4 3 lines from the bottom, the sentence starting "The Brd2 and Tcf712 alleles showed a similar, but less pronounced, conservation of phenotype.." requires clarification. Do they mean similar to Atp11a, to each other, or to both?

I have read this submission. I believe that I have an appropriate level of expertise to confirm that it is of an acceptable scientific standard.

## References

[1] LekMKarczewskiKJMinikelEVSamochaKEBanksEFennellTO'Donnell-LuriaAHWareJSHillAJCummingsBBTukiainenTBirnbaumDPKosmickiJADuncanLEEstradaKZhaoFZouJPierce-HoffmanEBerghoutJCooperDNDeflauxNDePristoMDoRFlannickJFromerMGauthierLGoldsteinJGuptaNHowriganDKiezunAKurkiMIMoonshineALNatarajanPOrozcoLPelosoGMPoplinRRivasMARuano-RubioVRoseSARuderferDMShakirKStensonPDStevensCThomasBPTiaoGTusie-LunaMTWeisburdBWonHHYuDAltshulerDMArdissinoDBoehnkeMDaneshJDonnellySElosuaRFlorezJCGabrielSBGetzGGlattSJHultmanCMKathiresanSLaaksoMMcCarrollSMcCarthyMIMcGovernDMcPhersonRNealeBMPalotieAPurcellSMSaleheenDScharfJMSklarPSullivanPFTuomilehtoJTsuangMTWatkinsHCWilsonJGDalyMJMacArthurDG: Analysis of protein-coding genetic variation in 60,706 humans. *Nature*.2016;536(7616) : 10.1038/nature19057 285-91 10.1038/nature19057 27535533PMC5018207

# Response

**Published:** 2017-03-06

**Authors:** Tim Mohun

**Affiliations:** The Francis Crick Institute, UK

The sentence on p4 referenced in the reviewer's comment describes a trend in the similarity of phenotypes across all of the embryos within a particular mutant line. So we are not comparing the phenotypes between lines, but whether there is consistency between different embryos within any individual line.
